# Application of CRISPR/Cas9-Based Gene Editing in HIV-1/AIDS Therapy

**DOI:** 10.3389/fcimb.2019.00069

**Published:** 2019-03-22

**Authors:** Qiaoqiao Xiao, Deyin Guo, Shuliang Chen

**Affiliations:** ^1^School of Basic Medical Sciences, Institute of Medical Virology, Wuhan University, Wuhan, China; ^2^Laboratory of Medical Virology, School of Medicine, Sun Yat-sen University, Guangzhou, China; ^3^Department of Veterinary Biosciences, Center for Retrovirus Research, Ohio State University, Columbus, OH, United States

**Keywords:** HIV-1/AIDS, CRISPR/Cas9, gene editing, host factors, latent viral reservoirs

## Abstract

Despite the fact that great efforts have been made in the prevention and therapy of HIV-1 infection, HIV-1/AIDS remains a major threat to global human health. Highly active antiretroviral therapy (HAART) can suppress virus replication, but it cannot eradicate latent viral reservoirs in HIV-1/AIDS patients. Recently, the clustered regularly interspaced short palindromic repeat (CRISPR)/CRISPR-associated nuclease 9 (Cas9) system has been engineered as an effective gene-editing technology with the potential to treat HIV-1/AIDS. It can be used to target cellular co-factors or HIV-1 genome to reduce HIV-1 infection and clear the provirus, as well as to induce transcriptional activation of latent virus in latent viral reservoirs for elimination. This versatile gene editing technology has been successfully applied to HIV-1/AIDS prevention and reduction in human cells and animal models. Here, we update the rapid progress of CRISPR/Cas9-based HIV-1/AIDS therapy research in recent years and discuss the limitations and future perspectives of its application.

## Introduction

Human immunodeficiency virus/acquired immunodeficiency syndrome (HIV/AIDS) is still a severe health problem worldwide. According to the statistics from World Health Organization (WHO) (http://www.who.int/hiv/en/), about 36.9 million people were living with HIV by the end of 2017, with about 1.8 million newly infected individuals. Interestingly, only 59% of infected patients received highly active antiretroviral therapy (HAART), which is still the main therapeutic strategy for HIV-1 patients and has reduced the morbidity and mortality of HIV-related disease (Palella et al., 1998). However, HAART cannot efficiently eliminate latent viral reservoirs, making HIV-1/AIDS a chronic and incurable disease (Chun et al., 1997b; Lorenzo-Redondo et al., 2016; Huang et al., 2018). Moreover, the high costs of therapy, side effects, and drug resistance should also be considered in HIV-1/AIDS treatment (Larder et al., 1989). Therefore, new therapeutic strategies to inhibit HIV-1 replication and eliminate latent reservoirs are urgently needed.

In the recent years, the three main nuclease-mediated gene editing tools including transcription activator-like nucleases (TALENs), zinc finger nucleases (ZFNs), and clustered regularly interspaced short palindromic repeat (CRISPR)/CRISPR-associated nuclease 9 (Cas9) have been widely used in HIV-1/AIDS treatment researches (Tebas et al., 2014; Yin et al., 2018; Yu et al., 2018). A clinical trial with ZFN mediated C-C chemokine receptor type five (CCR5) editing in autologous CD4 T cells had been successfully conducted in HIV-1 infected patients, which demonstrated ZFNs-CCR5 modification may be effective and safe in human AIDS therapy (Tebas et al., 2014). Due to the costly and time-consuming construction of TALENs and ZFNs (Khalili et al., 2015), the newest gene-editing technique CRISPR/Cas9 has been developed rapidly with the advantages of precise insertion, deletion, and replacement of target DNA sequences (Hsu et al., 2014). The first CRISPR/Cas9 application in the prevention of HIV-1 infection was performed in 2013 by disruption of latent HIV-1 provirus (Ebina et al., 2013). From then on, numerous studies on HIV-1/AIDS gene therapy by CRISPR/Cas9 technology have been reported (Wang et al., 2014, 2017; Ye et al., 2014; Hou et al., 2015; Kang et al., 2015; Li C. et al., 2015; Zhu et al., 2015; Liu et al., 2017; Xu et al., 2017; Chen et al., 2018; Qi et al., 2018), suggesting its tremendous potential to treat HIV-1/AIDS. In this review, we summarize studies concerning the principles, function, and application of CRISPR/Cas9 in HIV-1/AIDS treatment in the last few years, and highlight problems that need to be addressed in the future.

## Human Immunodeficiency Virus

AIDS is still a major global health problem. It is caused by HIV infection and induces immune destruction (Bowers et al., 2014). There are two different types of HIV, HIV-1, and HIV-2. They both have many similarities and both can lead to AIDS (Nyamweya et al., 2013). Compared to HIV-1, HIV-2 has lower transmissibility and is less pathogenic. HIV-1 is recognized as the major cause of AIDS and becomes the main target to prevent and cure AIDS.

**Figure 1:**
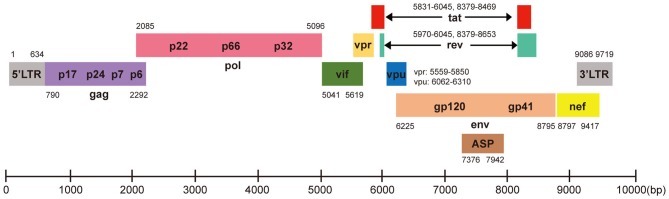
HIV-1 DNA genome structure for CRISPR-Cas9 targeting. HIV-1 genome is ~9.8 kb with 5′LTR, 3′LTR, and encodes 10 viral proteins. Each gene is showed in its specific location based on HXB2 (K03455). The Cas9 target sequences listed in Table 1.

**Figure 2:**
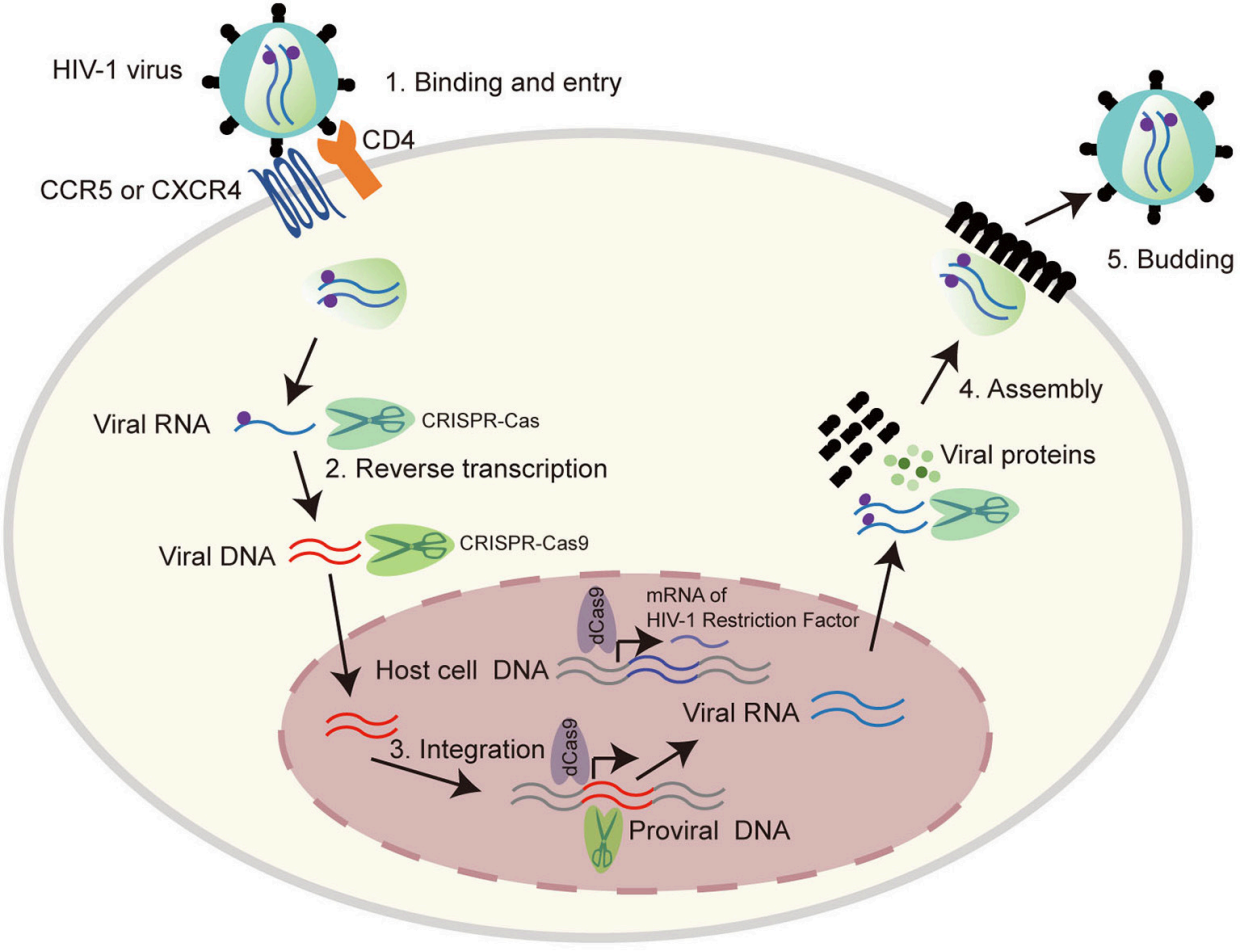
The life cycle of HIV-1 provides possible CRISPR-Cas9 targets. HIV-1 life cycle is carried out in six stages: (1) Binding and entry. HIV-1 invades into host cells by binding its gp120 to CD4 receptor on the cell surface, and then to the co-receptor CCR5 or CXCR4. This binding causes HIV-1 and cell membrane fusion, by which HIV-1 enters into host cells and releases its viral RNA. (2) Reverse transcription. HIV-1 RNA was reverse transcribed into double stranded DNA by reverse transcriptase. (3) Integration. The viral DNA enter into nucleus and integrate into host genomic DNA by integrase. (4) Replication and assembly. New viral RNA generated by proviral DNA can be used as genomic RNA to make viral proteins. These proteins combining with viral RNA moves to cell surface to form immature viral particles. (5) Budding. The immature viral particles are released from cells and produce viral protease which can break the long protein chain to form the mature virus. Many regulators play important roles in the life cycle of HIV-1, thus provide potential CRISPR-Cas9 targets as listed in Table 2.

HIV-1 is a retrovirus with an RNA genome of about 9.8 kb. The complete genome is flanked by two long terminal repeat (LTR) sequences and it encodes 10 viral proteins including gag, pol, vif, vpr, vpu, env, tat, rev, nef, and the antisense protein (ASP) (Cassan et al., 2016; Liu Z. et al., 2018), which have different functions in virus invasion and replication (Figure 1). Three main routes that spread HIV-1 are sex, intravenous injection, and vertical transmission (Hladik and McElrath, 2008; Cohen et al., 2011). All HIV-1 infected patients have three stages, acute HIV infection, chronic HIV infection (clinical latency), and clinical disease (AIDS) (Sharp and Hahn, 2011). Mechanistically, HIV-1 invades host cells by binding its gp120 envelope protein to the CD4 receptor on the membrane of the target cell, and then interacts with the CCR5 or CXCR4 coreceptor which depends on the tropism of viral strain. The host cells mainly correspond to T cells, monocytes and dendritic cells and even microglial cells, astrocytes, and perivascular macrophages in central nervous system. The life cycle of HIV-1 is complex, which contributes to ineffective virus elimination (Figure 2). HIV-1 entry into cells will establish two types of infection including latent infection and active infection. Latent infection occurs in the early stage of infection within a few cells, while active infection appears in most cells (Chun et al., 1997a,b, 1998; Chavez et al., 2015). For active infection, the provirus is active and produces viral particles which make infected cells bud new progeny virions. The establishment of latent infection may be mediated by complex mechanisms, including RNA interference (Huang et al., 2007; Patel et al., 2014; Ruelas et al., 2015), chromatin environment (du Chene et al., 2007; Gallastegui et al., 2011), transcription factors (Barboric et al., 2001; Mojica et al., 2005; Lenasi et al., 2008), and HIV-1 provirus integration sites (du Chene et al., 2007; Sunshine et al., 2016). Latent infection results in the generation of latent reservoirs, which contain infected resting CD4^+^ T cells (Siliciano et al., 2003; Chun et al., 2008), astrocytes (Churchill et al., 2006; Narasipura et al., 2014), macrophages (Smith et al., 2003), and microglial cells (Bagasra et al., 1996; Nath, 2015). The latent reservoirs are often located in gastrointestinal tracts (Smith et al., 2003; Chun et al., 2008), brains (Bagasra et al., 1996; Fischer-Smith et al., 2001), and lymphoid tissues (Chun et al., 2008), which are difficult to reach by antiviral drugs. Once the latently infected cells are reactivated by stimuli, newly generated virus will be produced and infect neighboring cells, then a new latent reservoir will re-establish. Therefore, HIV-1 latent reservoirs are the major challenge for an effective cure for HIV-1/AIDS.

## CRISPR/Cas9 Technology

**Figure 3:**
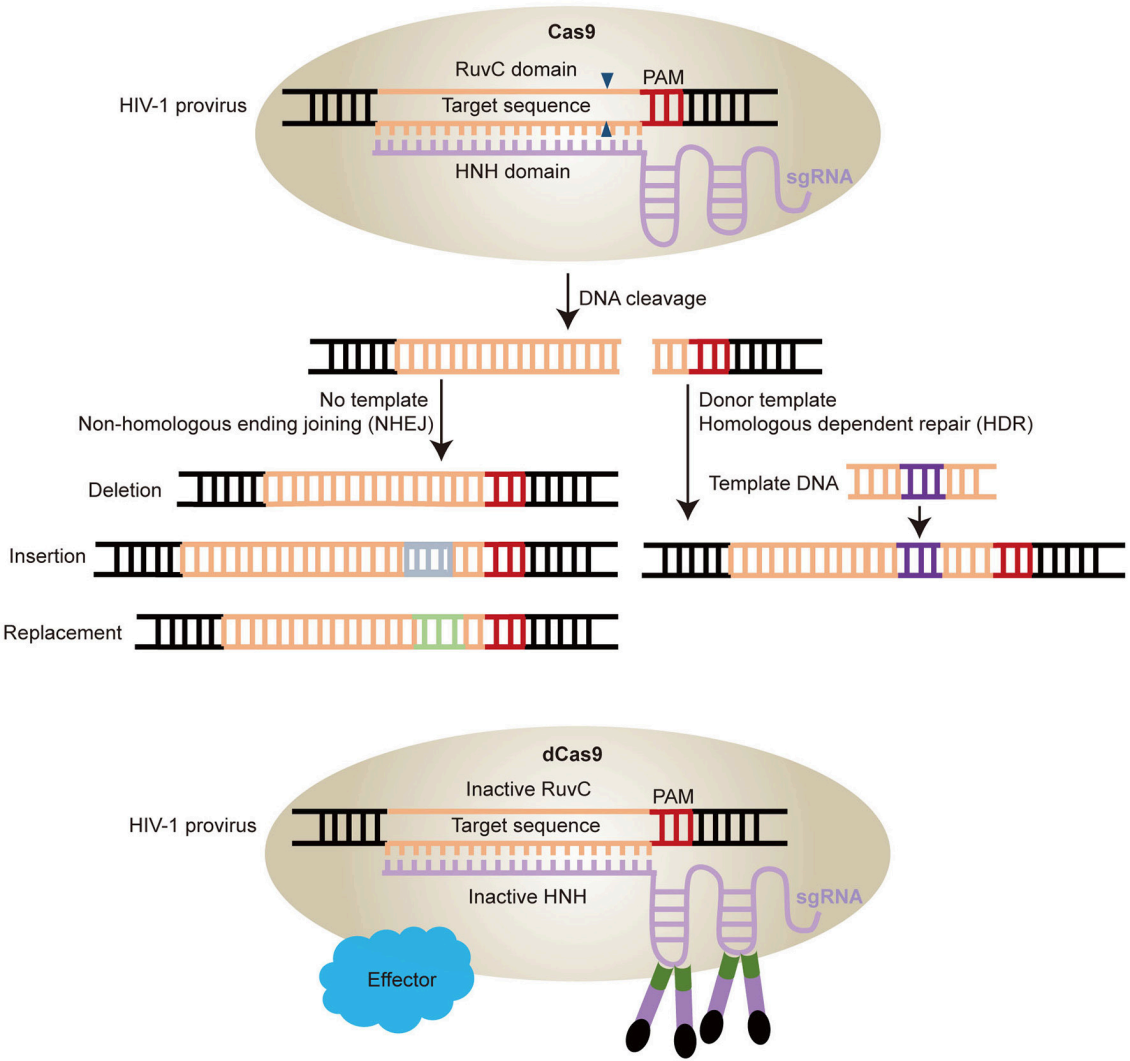
Schematic diagram of HIV-1 provirus DNA modification by CRISPR/Cas9 technology. **(A)** Cas9 protein combined with sgRNAs can induce gene editing at specific sites. The double stranded breaks are repaired by two pathways. One is Non-homologous ending joining (NHEJ) with no template, which will induce deletion, replacement, and insertion. The other is Homologous dependent repair (HDR) with donor templates. **(B)** Cas9 can be engineered into catalytically inactive Cas9 (dCas9) by mutations of two nuclease domains RuvC and HNH. The dCas9 can be fused with various effectors with a site-specific DNA-binding domain to activate the latent virus by sgRNAs target the HIV-1 LTR region.

CRISPR repetitive sequences were first observed in 1987 (Ishino et al., 1987) and were later shown to derive from conjugative plasmids and bacteriophages and act as a mechanism of adaptive immunity (Mojica et al., 2005, 2009; Barrangou et al., 2007). Breakthrough research was made by Jinek et al. that showed a DNA endonuclease Cas9 guided by two RNAs could introduce target DNA cleavage *in vitro* (Jinek et al., 2012). From then on, CRISPR/Cas9 technology was developed rapidly and achieved great progress in the field of gene therapy in human CD34^+^ hematopoietic stem and progenitor cells (HSPCs) (De Ravin et al., 2017; Niethammer et al., 2018). The CRISPR-Cas9 apparatus, involves the Cas9 helicase which can bind to RNA transcribed from the palindromic repeats of host DNA and cleave invasive DNA paired with RNA spacers, a transcript from the short stretches of host DNA acquired from extra chromosomal elements. In this system, the transcript of palindromic repeats of DNA named the trans activating CRIPSR RNA (tracr RNA), while the spacers' transcript called CRIPSR RNA (crRNA). The tracr RNA and crRNA can be linked together to form a single guide RNA (sgRNA) that can direct Cas9 to induce DNA cleavage in the Protospacer adjacent motifs (PAMs) region (Jinek et al., 2012; Cong et al., 2013; Hsu et al., 2014). The Cas9 nuclease has two activity domains histidine-asparagine-histidine (HNH) and RuvC, cleaving different DNA strands (Jinek et al., 2014; Nishimasu et al., 2014). The sgRNA base-pairs with the target strand, which is cleaved by the HNH domain, while the non-target strand is cleaved by the RuvC domain (Figure 3A) (Jinek et al., 2012). The cleavage induced double stranded DNA break (DSB) will be repaired by non-homologous end-joining (NHEJ) in the absence of template or homology-directed repair (HDR) with homologous template such as double strand DNA (dsDNA) and single strand DNA (ssDNA) (Figure 3A). If the donor DNA delivered to the cell has homology arms flanking the locus targeted by Cas9, it will be used as a template for repair and therefore introduces the desired substitution sequence (Ran et al., 2013b; Hsu et al., 2014). However, for the NHEJ, it will randomly introduce insertion, deletion, and replacement (Figure 3A) (Ran et al., 2013b).

CRISPR/Cas9 technology can not only mediate gene editing effectively, but also confer biological functions. Mutation of Cas9 in two nuclease domains induces inactive Cas9 (dCas9), which is used as locus-specific DNA-binding protein (Figure 3B) (Gilbert et al., 2013; Qi et al., 2013). This dCas9 fused with a transcription activator or repressor domain can regulate gene expression (Figure 3B) (Konermann et al., 2015). Moreover, other gene editing technologies, such as CRISPR/Cpf1, a single RNA-guided endonuclease without tracrRNA, and CRISPR-Cas13a/C2c2, a RNA-guided RNA-targeting effector that induces ssRNA cleavage, have been developed based on the foundation of CRISPR/Cas9 technology (Zetsche et al., 2015; Gootenberg et al., 2017). Therefore, CRISPR/Cas9 is a powerful gene editing tool with unlimited potential in biomedical research field.

## CRISPR/Cas9 Technology Application in HIV-1/AIDS Treatment

CRISPR/Cas9 technology has been widely applied in HIV-1/AIDS research using experimental laboratory adapted HIV-1 strains in the past few years because of its characteristics of simple, high efficiency, and limited off-target effect (Duan et al., 2014). Its targets include HIV-1 genome (Table 1) and host factors (Table 2).

**Table 1:** CRISPR/Cas9 systems target HIV-1 provirus for excision and elimination.

**CRISPR-Cas**	**Delivery**	**Target region**	**Cell type/organism**	**Targeting locus**	**Target sequence**	**Efficiency**	**References**
SpCas9	Transfection	LTR (U3 region)	293T, Hela, Jurkat	465–484	GTTAGACCAGATCTGAGCCT	30–90%	Ebina et al. (2013)
SpCas9	Transfection	LTR (U3 region)	CHME5, TZM-Bl, U937	101–127	GCCAGGGATCAGATATCCACTGACCTT	30–90%	Hu et al. (2014)
				312–341	GAGTACTTCAAGAACTGCTGACATCGAGCT		
SpCas9	Lentivirus	LTR (R region)	293T-CD4-CCR5, 293 Primary T cells, hPSC	464–486	GGTTAGACCAGATCTGAGCCTGG	20–90%	Liao et al. (2015)
				485–507	GGGAGCTCTCTGGCTAACTAGGG		
SpCas9	Transfection	Rev (the second exon)	JLat10.6	8513–8532	GGTGGTAGCTGAAGAGGCAC	30%	Zhu et al. (2015)
SpCas9	Lentivirus	Gag/Pol/Rev/Env	SupT1	2249–2277	TCAGATCACTCTTTGGCAGCGAC	30–90%	Wang Z. et al. (2016)
				8497–8525	GTGCCTCTTCAGCTACCACCGCT		
SpCas9	Lentivirus	LTR (U3 and R region)	J.Lat FL,SupT1	300–408	GCCACTCCCCAGTCCCGCCC	35–98%	Lebbink et al. (2017)
				463–482	GCTCAGATCTGGTCTAACCA		
SaCas9	Lentivirus AAV	LTR and gag (U3 region)	Tg26 transgenic mouse	83–103	GCAGAACTACACACCAGGGCC	20–80%	Yin et al. (2017)
				380–399	GTGTGGCCTGGGCGGGACTG		
				1061–1081	GGATAGATGTAAAAGACACCA		
SaCas9	Lentivirus	LTR (U3 region)	TZM-Bl, C11	289–309, 9364–9384	ACATGGCCCGAGAGCTGCATC	20–60%	Wang Q. et al. (2018)
				379–399, 9454–9474	GGTGTGGCCTGGGCGGGACTG		

**Table 2:** CRISPR/Cas9 systems target co-receptor CCR5 or CXCR4 and restriction factors.

**CRISPR-Cas9**	**Delivery**	**Target gene/restriction factors**	**Cells**	**Strategy**	**Target sequence**	**Efficiency (%)**	**References**
SpCas9	Transfection	CCR5	K562	Disruption	TGACATCAATTATTATACAT	13	Cho et al. (2013)
SpCas9	Transfection	CCR5	iPSC	CCR5Δ32	GATACAGTCAGTATCAATTC	33–100	Ye et al. (2014)
SpCas9	Lentivirus	CCR5	TZM-Bl, CEMss,	Disruption	GCTTGTGACACGGACTCAAG GGTCCTGCCGCTGCTTGTCA GTAAACTGAGCTTGCTCGCT	10.8–67.7	Wang et al. (2014)
SpCas9	Lentivirus Adenovirus	CCR5	TZM-Bl, CHO, C8166, primary CD4^+^ T	Disruption	TCACTATGCTGCCGCCCAGT CAATGTGTCAACTCTTGACA	32–75	Li C. et al. (2015)
SpCas9	Transfection	CCR5	iPSC	Disruption	TGACATCAATTATTATACAT CATACAGTCAGTATCAATTC GACATTAAAGATAGTCATCT	12.5–30.8	Kang et al. (2015)
SpCas9	Transfection	CCR5	K562, CD34^+^ HSPC	Disruption	ACTGGGCGGCAGCATAGTGA CCCAGAAGGGGACAGTAAGA	19–46	Xu et al. (2017)
SpCas9	Lentivirus	CCR5	Jurkat, primary CD4^+^ T	CCR5Δ32	ACAGTCAGTATCAATTCTGG GACATTAAAGATAGTCATCT	40–60	Qi et al. (2018)
SpCas9	Lentivirus	CXCR4	Ghost, Jurkat, primary CD4^+^ T	Disruption	GCTTCTACCCCAATGACTTG GTTCCAGTTTCAGCACATCA	10–45	Hou et al. (2015)
SaCas9	Lentivirus AAV	CXCR4	TZM-Bl, Ghost, Jurkat, primary CD4^+^ T	Disruption	CCTGGTATTGTCATCCTGTCC TCCTGCTATTGCATTATCATC	8.5–80	Wang et al. (2017)
SpCas9	Lentivirus	CCR5 and CXCR4	TZM-Bl, Jurkat, primary CD4^+^ T	Disruption	GCTTCTACCCCAATGACTTG GTTCCAGTTTCAGCACATCA CATACAGTCAGTATCAATTC	15–40.5	Liu et al. (2017)
SpCas9	Lentivirus	Restriction factors	293T, CEMss	APOBEC3G(A3G) and APOBFC3B(A3B)	–	50–90	Bogerd et al. (2015)
–	–	Restriction factors	-	SERINC, HUSH, NONO	–	–	Rosa et al. (2015); Gonzalez-Enriquez et al. (2017); Chougui et al. (2018); Lahaye et al. (2018); Yurkovetskiy et al. (2018)

### Inactivation and Elimination of HIV-1 Provirus by CRISPR/Cas9 Technology

The main obstacle for HIV/AIDS treatment is efficient targeting of latent viral reservoirs. HAART can control HIV-1 replication in patients, but it cannot completely eliminate the provirus in resting CD4^+^ T cells, resulting in the stability of latent viral reservoirs for many years (Siliciano et al., 2003). In early studies, researchers used Tre-recombinases to specifically target HIV-1 LTR, which resulted in the excision of HIV-1 provirus in HeLa cells (Sarkar et al., 2007). Qu et al. also generated ZFNs to target TAR region in HIV-1 LTR, which led to efficient cleavage of integrated HIV-1 proviral DNA in Jurkat T cells and the latent cell line C11 (Qu et al., 2013).

The CRISPR/Cas9-based approach was first tested in HIV-1/AIDS treatment in 2013. Ebina et al. successfully used CRISPR/Cas9 to suppress the expression of HIV-1 genes in Jurkat cell lines by targeting HIV-1 LTR (Ebina et al., 2013). The target sites were the NF-κB binding cassettes located in the U3 region of LTR and TAR sequences in R region, respectively. This resulted in efficient inhibition of HIV-1 provirus transcription and replication (Ebina et al., 2013). More importantly, it also showed that CRISPR/Cas9 could eliminate internal integrated viral genes from the infected host cell chromosome, which suggested that CRISPR/Cas9 may be a potential tool for HIV-1/AIDS treatment (Ebina et al., 2013). Soon afterwards, research on excision of HIV-1 genome via CRISPR/Cas9 was conducted by Hu et al. (2014). They used Cas9/gRNA to target conserved sites in HIV-1 LTR U3 region, resulting in inactivating viral gene expression and restricting virus replication in a HIV-1 latently infected T cell line, pro-monocytic cell line, and microglial cell line with little genotoxicity, and no detectable off-target editing (Hu et al., 2014). Liao et al. also demonstrated that targeting multiple sites of the HIV-1 genome could increase the efficiency of excision and disruption of non-integrated proviral genome (Liao et al., 2015). In addition, combination of two effective sgRNAs to target different regions of the HIV genome could prevent viral replication and escape (Lebbink et al., 2017). Recently, Wang et al. also demonstrated that Staphylococcus aureus Cas9 (SaCas9)/gRNAs in an all-in-one lentiviral vector could excise latent HIV-1 provirus and suppress provirus reactivation (Wang Q. et al., 2018). Furthermore, the combined SaCas9/gRNAs showed higher efficiency in disrupting HIV-1 genome than single sgRNA mediated SaCas9 editing (Wang Q. et al., 2018).

Mutational inactivation of HIV-1 provirus by a single sgRNA induced CRISPR/Cas9 editing has also been reported (Wang G. et al., 2018). When targeting the LTR sequence and essential genes for viral replication by a single sgRNA, the HIV-1 provirus was inactivated by mutation of the target site. However, the virus can escape from a single gRNA mediated cleavage. This viral breakthrough can be alleviated by a combinatorial CRISPR/Cas9 gene-editing approach (Lebbink et al., 2017). Zhu et al. used CRISPR/Cas9 target LTR of provirus in HIV-1 latently infected T cell lines. Sequencing analysis demonstrated mutations occur at each target site, resulting in reduction of viral gene expression and viral production while on tumor necrosis factor alpha (TNFα) treatment (Zhu et al., 2015). Several studies also performed targeting on incoming virus by CRISPR/Cas9 system. They confirmed that dual sgRNAs have higher cleavage efficiency on a non-integrated HIV-1 reporter plasmid than a single sgRNA (Wang et al., 2016a; Lebbink et al., 2017). Recent work also suggested that CRISPR/Cas9 could cleave the non-integrated HIV-1, resulting in a 3–4-fold reduction of integrated HIV-1 provirus. Surprisingly, the NHEJ-mediated DNA repair mechanism also responds for the non-integrated HIV-1 provirus (Mefferd et al., 2018). Thus, the CRISPR/Cas9 functions in both HIV-1 and proviral DNA in latent cells, of which makes it more potential and promising in HIV-1/AIDS treatment.

Excision of HIV-1 proviral DNA in animal models was reported in 2017 (Yin et al., 2017). Researchers demonstrated the feasibility and efficiency of disrupting HIV-1 provirus using an all-in-one adeno-associated virus (AAV) combined with multiplex sgRNAs and SaCas9 in three different animal models. Quadruplex sgRNA/SaCas9 AAV-DJ/8 intravenously injected into Tg26 mice could cleave HIV-1 proviral DNA and significantly reduce virus replication. Additionally, after intravenous injection of quadruplex sgRNA/SaCas9 AAV-DJ/8 in humanized bone marrow/liver/thymus (BLT) mice infected with HIV-1, the cleavage of provirus was detected in brain, colon, spleen, heart, and lung. This system could also inhibit HIV-1 replication in Eco-HIV acutely infected mice (Yin et al., 2017). This successful application of excision and elimination of HIV-1 proviral DNA by SaCas9/gRNA *in vivo* delivered via AAV lays the foundation for the design of clinical trials in humans.

### Disruption of Co-receptors CCR5 and CXCR4 by CRISPR/Cas9 Technology

In addition for targeting HIV-1 genome, CRISPR/Cas9 technology can also be used to block HIV-1 entry by editing of co-receptors. HIV-1 enters host cells through binding to the CD4 receptor and CCR5 or CXCR4 co-receptors (Cocchi et al., 1995). As CD4 is critical for functional immune system, disruption of CD4 is an unadvisable strategy in preventing HIV-1 infection. It has been reported that individuals with a homozygous 32-bp deletion of the *CCR5* gene (*CCR5*Δ*32*) could live healthily and resist R5-tropic HIV-1 infection naturally (Samson et al., 1996; Biti et al., 1997). Transplantation of *CCR5*Δ*32* HSPCs to a “Berlin Patient” who experienced with acute myeloid leukemia (AML) and HIV-1 infection accidently prevented HIV-1 replication and rebound so far (Hutter et al., 2009; Allers et al., 2011). This is an encouraging case, but due to the few homozygous *CCR5*Δ*32* donors, broad application of this strategy to HIV-1/AIDS patients is significantly limited (Michael et al., 1997; Zimmerman et al., 1997). Therefore, co-receptors CCR5 and CXCR4 become the potential targets for HIV-1/AIDS gene therapy. Gene editing of CCR5 by ZFNs has been successfully used in CD4^+^ T cells and CD34^+^ HSPCs, which could resist HIV-1 infection (Perez et al., 2008; Holt et al., 2010). Tebas et al. conducted disruption of CCR5 via ZFN delivered by adenoviral vector in CD4^+^ T cells from HIV patients and reinfused autologously after *ex vivo* expansion, leading to significant decrease of HIV DNA and RNA to undetectable level in most patients (Tebas et al., 2014). This important clinical trial (NCT00842634) was followed by additional studies showing that CCR5-modified cells could be detected in HIV-1 patients for several months (Tebas et al., 2014). For the other co-receptor, CXCR4 could also be modified by ZFN to resist to X4-tropic HIV-1 infection (Wilen et al., 2011). While, simultaneous editing of CXCR4 and CCR5 by ZFN in Sup T1-R5 and primary CD4^+^ T cells was also reported (Didigu et al., 2014). Interestingly, the *in vivo* study showed a significant survival advantage in the presence of HIV-1 infection in NSG mouse transferred with modified primary CD4^+^ T cells compared to the unmodified cohort (Didigu et al., 2014).

Compared with ZFN technology, the CRISPR/Cas9 approach provides appropriate target sites with simple design and plasmid construction. CRISPR/Cas9 has been widely used in disrupting CCR5 and CXCR4 expression. In 2013, Cho et al. showed that silencing of CCR5 by CRISPR/Cas9 can be successfully achieved in human embryonic kidney (HEK) 293T cells by transfecting Cas9 and sgRNAs (Cho et al., 2013). Soon after that, in 2014, Ye et al. conducted a combined CRISPR/Cas9 or TALENs with the piggyBac technology to perform the homozygous *CCR5*Δ*32* mutation in induced pluripotent stem cells (iPSCs) seamlessly. These CCR5-modified iPSCs could normally differentiate into monocytes/macrophages, which were resistant to HIV-1 infection (Ye et al., 2014). In 2015, Li et al. used adenovirus-delivered CRISPR/Cas9 combined with sgRNAs targeting the fourth exon of CCR5 to disrupt CCR5 expression. Two specific sgRNAs can induce more than 60% cutting efficiency in TZM-BL cells. The results were also confirmed in Chinese hamster ovary (CHO) and human T cell lines. Finally, they used a chimeric Ad5/F35 adenovirus vector to deliver CRISPR/Cas9 system to human CD4^+^ T cells to silence CCR5 expression, which protected cells from HIV-1 infection with high efficiency and little off-target effects (Li C. et al., 2015). In 2017, Xu et al. used CRISPR/Cas9 system to target CCR5 gene in human CD34^+^ HSPCs and achieved long-term CCR5 disruption *in vivo*, which resulted in inhibition of HIV-1 infection. Silencing of CCR5 expression was stable in the secondary repopulating hematopoietic stem cells (HSCs), which provides a basis for the future of developing an HIV-1/AIDS cure for clinical application by transplanting CCR5-modified HSCs (Xu et al., 2017).

Meanwhile, several studies have reported CXCR4 gene disruption by CRISPR/Cas9. Hou et al. efficiently disrupted CXCR4 expression in human CD4^+^ T cells and rhesus macaque CD4^+^ T cells using CRISPR/Cas9 delivered by lentivirus and two sgRNAs that specifically target CXCR4 conserved sequences. Reduced p24 production was observed in the human CXCR4 knockout (KO) CD4^+^ T cells after infection with X4-tropic HIV-1, which suggested that CXCR4-modified cells resist HIV-1 infection (Hou et al., 2015). Moreover, CXCR4-edited CD4^+^ T cells by CRISPR/Cas9 behaved normally in propagation and there was no detectable off-target effect and toxicity in CD4^+^ T cells (Hou et al., 2015). Other researchers also disrupted CXCR4 expression by Cas9/gRNA ribonucleoprotein (Cas9 RNPs) in human CD4^+^ T cells, which reduced about 40% of CXCR4 expression on the cell surface (Schumann et al., 2015). When provided with repair template, the cellular repair machineries could perform the desired modification in human T cells. Although the CRISPR-Cas9 induced NHEJ process was a stochastic event, the deep sequencing revealed the knock-in efficiency up to 20% (Schumann et al., 2015). Further, a *Staphylococcus aureus*(SaCas9), which is 1 kb shorter compared to SpCas9, was used to disrupt CXCR4 in human primary CD4+ T cells delivered by AAV and protected cells from HIV-1 infection with low toxicity and little off-target effects (Wang et al., 2017). As CXCR4 and its ligand CXC12 (SDF-1) play pivotal roles in hematopoietic/progenitor cells development and thymic differentiation (Nagasawa et al., 1996; Dar et al., 2006), we should consider the safety and side effects of clinical application by targeting CXCR4 in HSPCs. However, CXCR4-deficient human T cells remain functional in a mouse model (Chung et al., 2010; Yuan et al., 2012), and we also used CRISPR-Cas9 and piggyBac recombinant technologies to create CXCR4 P191A mutant with HIV-1 infection inhibition function and without deficiency of CXCR4 function(Liu S. et al., 2018), it is possible for CRISPR-Cas9 editing of CXCR4 in human mature post-thymic CD4+ T cells for the purpose of HIV-1/AIDS therapy.

### Reactivation of Latent HIV-1 Virus by CRISPR/Cas9 Technology

To eradicate latent HIV-1 reservoirs, it is necessary to reactivate dormant virus in the host cells and induce cell killing by HAART and activation of antiviral immune responses. This strategy is known as “shock and kill” (Barboric et al., 2001; Huang et al., 2007; Lenasi et al., 2008; Gallastegui et al., 2011; Archin et al., 2012; Rasmussen et al., 2014; Ruelas et al., 2015; Delagreverie et al., 2016; Kim Y. et al., 2018). Several drugs have been showed to reactivate viral gene expression, such as the histone deacetylase (HDAC) inhibitor (Manson McManamy et al., 2014; Walker-Sperling et al., 2016), which introduces the acetylation and remodeling of chromatin, thus resulting in the enhancement of HIV-1 RNA expression in the latent reservoirs (Archin et al., 2012). The drugs only induce the transcription of latent HIV-1 in cells, but do not kill the virus or cause cell death (Kim Y. et al., 2018). The combination use of latent reversing agents (LRAs) might overcome several side effects in patients under HAART and lead to a better efficiency of purging the HIV-1 latent reservoir (Bullen et al., 2014; Hill et al., 2014; Panfil et al., 2018). However, this approach may not be able to target all viral reservoirs, and therefore may not be much efficient (Ho et al., 2013; Rasmussen et al., 2014). Moreover, given the important side effects observed in patients treated with HAART and/or HDAC inhibitors, additional strategies to reactivate HIV-1 reservoirs need to be developed.

The CRISPR/Cas9 technology may be a potential tool for the activation of latent HIV-1 viral reservoirs. Many researchers have used deficient Cas9 (dCas9) fusion protein combined with sgRNAs specific to effector domains of target DNA sequences to activate or repress gene transcription (Gilbert et al., 2013, 2014; Sander and Joung, 2014; Konermann et al., 2015). The catalytically inactive dCas9 fused with transcription activator domains can activate viral gene expression in HIV-1 latent reservoirs, which may improve the “shock and kill” strategy (Zhang et al., 2015; Bialek et al., 2016; Ji et al., 2016; Limsirichai et al., 2016; Saayman et al., 2016; Kim et al., 2017). Zhang et al. designed 20 sgRNAs to target the LTR-U3 region of HIV-1 promoter and screened two target sites located near or at NF-κB binding sites with high specificity and efficiency (Zhang et al., 2015). Those specific target sgRNAs could induce reactivation of HIV-1 provirus in HIV-1 latent cell lines such as TZM-Bl epithelial cells, CHME5 microglial cells, and Jurkat T lymphocytic cells (Zhang et al., 2015). They also found that this reactivation could induce suicide death in CHME5 microglial cells and Jurkat T lymphocytic cells but not in TZM-Bl due to the accumulation of toxic viral proteins, without any effect on the non-reactivated cells (Zhang et al., 2015). Saayman et al. also designed 23 sgRNAs to target the LTR U3 region of HIV-1 provirus and found the robust activation sites also near NF-κB binding sequences. This activation system worked more efficiently than latency reversing compounds such as SAHA and prostratin in different latent T cells models (Saayman et al., 2016). Limsirichai et al. designed 7 sgRNAs to target the key functional elements of HIV-1 LTR including U3 region, NF-κB and Sp-1 binding sites, R domain, and U5 region (Limsirichai et al., 2016). All 7 sgRNAs could induce gene activation from HIV-1 LTR promoter and only 2 sgRNAs, overlapping with the NF-κB binding sites and transactivation response elements significantly stimulated latent HIV-1 gene expression (Limsirichai et al., 2016). Moreover, combining latency breaking reagents, including prostratin and SAHA, with CRISPR activators could increase latent HIV-1 re-activation (Limsirichai et al., 2016). Therefore, CRISPR/Cas9-mediated activation of viral transcription may provide an alternative approach to target and activate viral gene expression in latent HIV-1 reservoirs.

### Reactivation of Host Restriction Factors During HIV-1 Infection

Several proteins in mammalian cells function as restriction factors during infection by HIV-1 and other viruses (Chemudupati et al., 2019). However, these host factors are often weakly expressed in infected cells. Simultaneous activation of the expression of restriction factors may represent an alternative strategy to prevent HIV-1 replication. Borgerd *et al* used a Cas9-based approach to induce the expression of the restriction factors APOBEC3G (A3G) and APOBFC3B (A3B) in human cells (Bogerd et al., 2015). They also found that using two sgRNAs had higher efficiency than single sgRNA, and both activated proteins could block Vif-deficient HIV-1 infection by inducing dC residues to dU residues (dC-to-Du) editing of HIV-1 genome (Bogerd et al., 2015). However, studies on the application of CRISPR/Cas9 technology in activating cellular host factors to inhibit HIV-1 infection are very limited. Recently discovered restriction factors such as serine incorporator five (SERINC5) (Rosa et al., 2015; Gonzalez-Enriquez et al., 2017), human silencing hub (HUSH) (Chougui et al., 2018; Yurkovetskiy et al., 2018), and NONO (Lahaye et al., 2018) may be new targets to be considered for this application. SERINC5 inhibits virus infection by preventing virus and cell fusion (Gonzalez-Enriquez et al., 2017). The HIV-1 accessory protein Nef counteracts the function of SERINC5 by redirecting it to a Rab7-positive endosomal compartment, thus preventing its incorporation in newly generated virions (Rosa et al., 2015). The HUSH complex, composed by TASOR, MPP8, and periphilin, can be degraded by the viral protein Vpx via a DCAF1-dependent proteasomal pathway in primary T cells and HIV-2 infected cells (Chougui et al., 2018). Yurkovetskiy et al. also demonstrated that the HUSH complex can be degraded by Vpx and Vpr from HIV-1, HIV-2 and SIV to counteract HUSH-induced repression of provirus transcription (Yurkovetskiy et al., 2018). The two studies above suggested that the HUSH complex is a critical host factor in HIV infection. Lahaye et al. identified NONO as a capsid-binding factor for Cyclic GMP-AMP synthase (cGAS)-mediated immune activation in macrophages and dendritic cells after HIV-1/2 infection using a two-hybrid yeast screening (Lahaye et al., 2018). NONO directly interacted with HIV-1/2 capsid proteins to increase DNA sensing mediated by cGAS but had little effect on HIV infection, which suggest the important role of NONO in cGAS-mediated immune activation after infection with HIV (Lahaye et al., 2018). Since these representative restriction factors can inhibit HIV infection via different mechanisms, the CRISPR/Cas9 technology could be used to simultaneously activate their expression in infected cells in order to target different phases of the viral life cycle. This approach may provide new strategies for the treatment of HIV-1 infection, and more efforts are needed to further develop this toolset.

Activation of host restriction by CRISPR/Cas9 is usually delivered by lentivirus with high transduction efficiency and easy virus production. In addition, specific sgRNAs are essential for the activation of target gene. Two sgRNAs will enhance the expression of target gene by increasing the target specificity to assure the complete activation of gene promoter (Bogerd et al., 2015). As the restriction factors act on HIV-1 at different stages in the life cycle and the expression of some restriction factors is only induced by virus infection, better understanding of the molecular mechanisms of HIV-1 counteraction with restriction factors will provide more options and rationale for the design of CRISPR/Cas9. In addition, several restriction factors have dual functions in immunomodulation and different expression levels in various cells, which makes its more complex in the activation of gene expression by CRISPR/Cas9 (Chemudupati et al., 2019). Moreover, whether the enforced long-term activation of restriction factors expression has deleterious effects *in vivo* needs further investigation. For example, the virus restriction factor APOBEC3G, which is induced by IFNs, has an antiviral effect on HIV-1 and HBV (Wang et al., 2008). It has different expression levels in various cells. Whether its activated expression in different cells over a long period of time has deleterious effects *in vivo* has not been reported at present.

## Limitations of the CRISPR/Cas9 Application

CRISPR/Cas9 technology has been widely used not only in HIV-1/AIDS treatment (Chen et al., 2018), but also in other human diseases, such as frontotemporal lobar degeneration with tau inclusions (FTLD-tau) (Jiang et al., 2018), Parkinson's disease (PD) (Zhou et al., 2018) and Duchenne muscular dystrophy (DMD) (Lim et al., 2018),with the characteristics of safe, efficient and simple construction. However, some limitations must be considered before designing clinical trials.

One major concern is the potential off-target effect, which may induce important gene mutations and chromosomal translocations (Kimberland et al., 2018). Reduction of off-target effects is always of the greatest importance in clinical application. Some researchers had proved that the off-target cleavage mediated by Cas9 was very limited compared with other nucleases including ZFNs, TALENs, and homing endonucleases by ChIP-seq (Duan et al., 2014). However, the significant off-target phenomena were actually detected even for sgRNAs with six or more mismatches (Wang et al., 2015). Many efforts have been made to reduce off-target effects such as dimerization dependent RNA-guided FokI-dCas9 nucleases (RFNs) (Tsai et al., 2014), truncated guide RNAs (tru-gRNAs) (Fu et al., 2014), and paired Cas9 nickase (Ran et al., 2013a; Shen et al., 2014). Kaminski et al. modulated the CRISPR/Cas9 system by placing the Cas9 gene under the control of a minimal HIV-1 promotor, which is activated by viral transcriptional activator, Tat. This strategy makes Cas9 express in HIV-1 infectious cells and reduces the complications that caused by unnecessary high expression of Cas9 in cells (Kaminski et al., 2016). Otherwise, direct delivery of Cas9 RNPs rather than expressing plasmid to target cells will decrease the off-target effects. The Cas9 RNPs will be degraded after editing the target DNA, resulting in maximum on-target effects and minimum off-target effects. For example, the Cas9 RNPs have been used to disrupt CXCR4 in human primary CD4 T cells with undetectable off-target effects (Schumann et al., 2015). A recent report has also demonstrated that a RNP complex with R691A SpCas9 mutant introduces high efficient gene editing in human HSPCs with reduced off-target editing (Vakulskas et al., 2018). However, it has been showed that the application of RNPs in some kinds of cells can trigger innate immune responses, leading to cytotoxicity in cells. Chemical synthesis and phosphatase treatment of sgRNA to remove its 5'-ppp can inhibit innate immune responses and reduce cell death (Kim S. et al., 2018). Another recent report identified the preexisting humoral and cell-mediated adaptive immune responses to Cas9 in humans, of which should be taken in consideration before clinical trials (Charlesworth et al., 2019). As the immune response is determined by many factors such as the target tissue, the route of administration and the dose of Cas9 (Crudele and Chamberlain, 2018), the strategies about instantaneous expression of Cas9 need to be improved and developed, as well as the assessment of Cas9 immunogenicity before clinical trials should be conducted in the future.

Another major challenge in CRISPR/Cas9 application is how to effectively deliver this large complex into HIV-1 infected cells. According to previous reports, the main delivery vectors include adenoviral, lentiviral, and adeno-associated viral vectors (Wang et al., 2014; Hou et al., 2015; Li C. et al., 2015). Adenoviral vectors can be used in various CRISPR/Cas9 systems due to its capacity of incorporating large DNA fragments and its immunogenic effects in clinical trials have been improved (Wold and Toth, 2013). However, generation of recombinant adenoviral vectors could still represent an important limitation (Afkhami et al., 2016). Lentiviral vectors are widely used for delivering CRISPR/Cas9 systems into cells with high efficiency. It can integrate into the host genome and mediate stable expression, which will increase the risk of off-target effects (Wang et al., 2014; Khalili et al., 2017). Adeno-associated viral vectors have the characteristics of safety, low toxicity, and efficient delivery (Mingozzi and High, 2013). However, due to their small packaging size, they can only accommodate a small exogenous gene, and re-administration with the same virus induces immune responses and reduce efficacy of delivery and gene expression (Zaiss and Muruve, 2008; Mingozzi and High, 2013). One early report found that adeno-associated viral vectors could cause both cellular and humoral immune responses although it had various advantages in gene therapy (Mingozzi et al., 2009). These immune responses should be avoided by development of new AAV variants with different chemical modifications of AAV vector, immunological profiles and immunosuppression (Louis Jeune et al., 2013).

In addition for the viral vectors, cationic polymer polyethyleneimine (PEI) (Li L. et al., 2015), lipid-based reagents (Cardarelli et al., 2016), and nanoparticles-based approaches (Givens et al., 2018) have been also utilized to deliver the CRISPR/Cas9 system. To improve the delivery efficiency, researchers tried to combine the lipid nanoparticle captured mRNA of Cas9 with adeno-associated virus encoding a sgRNA and a repair template to mediate the correction of a hereditary tyrosinemia gene, fumarylacetoacetate hydrolase (FAH), in a mouse model (Yin et al., 2016). For the eradication of latent reservoirs in the central nervous system of HIV-1 infected patients, the major challenge of delivering the CRISPR/Cas9 system into brain is the blood brain barrier (BBB). The existence of the BBB will only allow limited lipophilic molecules and smaller molecules cross but block the transportation of large molecules. Although intracerebral injection and intracerebroventricular infusion strategies can overcome obstacles of BBB to cure brain diseases, the risk of brain damage makes its unoptimistic for human therapy. However, nanoparticle-based drug delivery to the brain may be an alternative strategy to overcome BBB. Various nanoparticles have been successfully used for brain target by receptor-mediated transcytosis, such as polymer nanoparticles (Fornaguera et al., 2015), magnetic nanoparticles (Nair et al., 2013), and gold nanoparticles (Mout et al., 2017). Many novel nanoparticles have been investigated as the potential carriers for delivering CRISPR/Cas9 system with less cytotoxic effect. In addition, different formulation of nanoparticles may have a predilection for specific organs including liver and lung (Givens et al., 2018). In conclusion, the potential of delivery CRISPR/Cas9 system by specific nanoparticles to target HIV-1 reservoirs in brains requires more investigation in the future. More recently, the Nanoblades delivery system, which involves murine leukemia virus-like particles (VLPs) and Cas9-sgRNA RNPs, has been confirmed to efficiently target EMX1 gene in human induced pluripotent stem cells (IPSCs) and Myd88 in human hematopoietic stem cells (HSCs), as well as *Fto* gene in mouse bone-marrow cells (BM). It can induce HDR in HEK293T cells by combination with a DNA template. It also can be engineered for transcriptional activation purpose if it is used for delivery of dCas9. In addition, the Nanoblades can be injected into the perivitelline space of mouse-zygotes to generate transgenic mice with mutations at *Tyr* gene. Moreover, *in vivo* editing of *Hpd* in the mice liver was achieved by injection of Nanoblades (Mangeot et al., 2019). Since the IPSCs and HSCs represent major interest for therapeutical applications, the Nanoblades has potential in HIV-1/AIDS treatment in the future. However, the off-target effect and safety of Nanoblades based editing of host factors, such as CCR5 and CXCR4, as well as HIV-1 genome should be tested in primary cells and animal models before application in clinical therapy.

CRISPR/Cas9 is considered as a potential antiviral tool, but HIV-1 has evolved escape mechanisms. Wang et al. found that Cas9/gRNA could inhibit HIV-1 replication, but soon the virus escaped from this inhibition due to NHEJ repair, which induced mutations around the cleavage sites (Wang et al., 2016b). Other researches also demonstrated that CRISPR/Cas9 could generate mutant viruses able to resist to Cas9/sgRNA by causing DNA repair in host cells (Wang Z. et al., 2016; Yoder and Bundschuh, 2016). To cope with this escape mechanism, Liang et al. put forward alternative solutions such as modifying sgRNA, reprogramming Cas9 nuclease and suppressing NHEJ activity (Liang et al., 2016). The negative findings of CRISPR/Cas9 make us more careful in designing sgRNAs and applying this technology for the treatment of HIV-1/AIDS in clinical trials.

## Conclusion

Currently, HAART is still the major strategy for treatment of HIV-1/AIDS patients in the clinic. It can reduce HIV-1 to an undetectable level and make AIDS a chronic disease. Recently, broadly neutralized antibodies showed promising results (Bar-On et al., 2018; Mendoza et al., 2018; Liu et al., 2019), but still have a long way to transfer from bench to bedside. With the development of gene editing technologies, such as ZFN, TALEN and CRISPR/Cas9, more and more recent work focuses on using these new strategies to eliminate the virus in patients. ZFN, with the size of ~1 kb, is easier to deliver. Nevertheless, the limitation of target site and high off-target effects make it difficult to be applied in the HIV-1/AIDS gene therapy field. TALEN is more flexible in DNA target design and has lower off-target effects compared with ZFN. However, the time-consuming and costly construction of the recognition site of TALEN for DNA target hampers the development of this gene editing tool. For CRISPR/Cas9, with more convenient and efficient design of target sites, less laborious vector construction, limited off-target effects, it can be applied quickly in every research field, not only in HIV-1/AIDS therapy. For different target DNA, it needs only a change of sgRNA to find the most effective site. Even though the large size of SpCas9 (~4.1 kb) decreases the efficiency of delivery, the alternative SaCas9 (~3.3 kb) will overcome this limitation to some extent. The CRISPR/Cas9 system is indeed a promising gene editing tool applied in gene therapy fields, however, the high target efficiency and effective delivery are essential for successful application in clinical trial. Moreover, the low off-target effect and safety must be the prerequisites of consideration.

The research concerning successful application of gene editing tools *in vitro* and mouse models to inhibit HIV-1 infection allows clinical trials to come true. ZFN had been used in clinical HIV-1/AIDS therapy and completed with promising results (NCT00842634, NCT01252641, and NCT01044654). Disruption of CCR5 by ZFN in autologous CD4^+^ T cells provided long-term HIV-1 resistant when reinfused these cells back into patients. Another clinical trial about ZFN gene-editing of CCR5 in HSPCs followed autologous engraftment have been conducted in 2015 (NCT02500849). This phase I study will continue to 2019 to estimate the potential of CCR5-disrupted HSPCs in HIV-1 resistance in AIDS patients. The CRISPR/Cas9 mediated clinical trial was first conducted in 2016. The modified human T cells were reinfused back into an individual with metastatic non-small cell lung cancer, which is supposed to have a promising result (Cyranoski, 2016). Therefore, gene editing of autologous HSPCs provides an option for resistance of HIV-1 infection in the future. For the successful editing of HSPCs by CRISPR/Cas9 in HIV-1/AIDS treatment, it is important to design and screen effective sgRNAs to reduce the off-target effects. The safety and delivery efficiency of CRISPR/Cas9 to HSPCs also need consideration, since long term expression of Cas9/sgRNA may induce non-specific injury to the host genome and immune response (Kaminski et al., 2016; Charlesworth et al., 2019). For the gene editing therapy in clinical treatment, the ethics of animal and human experimentation and the rationale are always the primary consideration before its application. Recently, the birth of gene-edited babies in 2018 has aroused widespread criticism around the scientific fields. It is the first time that the CCR5 gene edited human embryos by CRISPR-Cas9 were implanted to women to have HIV-1 resistant babies. This work would make a permanent change to the germ line, which could be passed on to the future generations. Obviously, this experiment failed to meet the ethical guideline concerning germline and embryo editing. In addition, it lacks the rationale to modify CCR5 in human embryos, since HAART can inhibit HIV-1 replication and the experimental couple can have a healthy baby. For HIV-positive mothers, cesarean section can protect babies from HIV infection. Even a HIV-positive father would have no risk to transmit to the babies. Moreover, a CCR5 edited baby cannot resist all HIV strains since the virus can evolved to utilize CXCR4 as alternative co-receptor. Finally, CRISPR/Cas9 technology has limitations in application such as off-target effects. The safety of heritable germline editing should be monitored and evaluated. The side effects of permanent editing of the *CCR5* gene have not been reported, yet some researches have showed that CCR5 deficiency increases the risk of symptomatic West Nile virus infection (Glass et al., 2006; Lim et al., 2008).

The major obstacle to cure HIV-1/AIDS is the existence of latent reservoirs. The “shock and kill” strategy is supposed to clear the HIV-1 reservoirs. Latency reversing agents (LRAs) have been used to reverse HIV-1 latency. However, the lack of specificity and the heterogeneous and dynamic nature of these drugs make this pharmacologic strategy less safe and inefficient (Darcis et al., 2018). CRISPR-based shock strategy with the characteristic of sequence specificity have advantages over pharmacological, but its potential drawbacks need to be evaluated, such as off-target effect and less efficient delivery method. The low metabolic activity in HIV-1 latently infected cells also inhibits the function of CRISPR/Cas9 reagents, however, the stimulation of specific cytokines and HIDAC inhibitors can enhance the CRISPR/Cas9 reagents mediated restricting of HIV-1 infection in latent cells.

CRISPR/Cas9 technology is a powerful gene editing tool and has been widely applied in experimental HIV-1/AIDS gene therapy researches. Moreover, it also has great potential to be applied in various areas such as medical genetic screening and gene ontology analysis (Xue et al., 2016). Its emergence brings the hope for 36.9 million individuals with HIV-1 infection, but it is worth noting that the negative effects such as off-target and viral escape must be considered. Therefore, successful cure of HIV-1/AIDS still has a long way to go. Clinical trials of CRISPR/Cas9 in HIV-1 treatment remains a challenge, and ethic must always be put in first place. The limitations and difficulties of this technology suggest that several aspects need to be improved for future applications: (1) Exploration of new vehicles to deliver CRISPR/Cas9 compound safely and effectively; (2) For activating the latent viral reservoirs by CRISPR/Cas9, several specific agents can be combined to enhance immune responses to eliminate the virus; (3) Design specific sgRNAs and explore new strategies to decrease off-target effects; (4) Understanding the exact mechanism of viral escape from the CRISPR/Cas9-induced effects is critical for the design of more effective strategies (5) Optimization of animal models of HIV-1/AIDS. The combination of the CRISPR/Cas9 technology with other strategies may help overcoming these limitations, thus leading to exciting and promising progress in the HIV-1/AIDS field.

## Author Contributions

QX and SC wrote the manuscript with input and edits from SC and DG. All authors approved it for publication.

### Conflict of Interest Statement

The authors declare that the research was conducted in the absence of any commercial or financial relationships that could be construed as a potential conflict of interest.

## References

[B1] Afkhami S. Yao Y. Xing Z. (2016). Methods and clinical development of adenovirus-vectored vaccines against mucosal pathogens. Mol. Ther. Methods. Clin. Dev. 3:16030. 10.1038/mtm.2016.30 27162933 PMC4847555

[B2] Allers K. Hutter G. Hofmann J. Loddenkemper C. Rieger K. Thiel E.. (2011). Evidence for the cure of HIV infection by CCR5Delta32/Delta32 stem cell transplantation. Blood 117, 2791–2799. 10.1182/blood-2010-09-309591 21148083

[B3] Archin N. M. Liberty A. L. Kashuba A. D. Choudhary S. K. Kuruc J. D. Crooks A. M.. (2012). Administration of vorinostat disrupts HIV-1 latency in patients on antiretroviral therapy. Nature 487, 482–485. 10.1038/nature11286 22837004 PMC3704185

[B4] Bagasra O. Lavi E. Bobroski L. Khalili K. Pestaner J. P. Tawadros R.. (1996). Cellular reservoirs of HIV-1 in the central nervous system of infected individuals: identification by the combination of in situ polymerase chain reaction and immunohistochemistry. AIDS 10, 573–585. 10.1097/00002030-199606000-00002 8780811

[B5] Barboric M. Nissen R. M. Kanazawa S. Jabrane-Ferrat N. Peterlin B. M. (2001). NF-kappaB binds P-TEFb to stimulate transcriptional elongation by RNA polymerase II. Mol. Cell. 8, 327–337. 10.1016/S1097-2765(01)00314-8 11545735

[B6] Bar-On Y. Gruell H. Schoofs T. Pai J. A. Nogueira L. Butler A. L.. (2018). Safety and antiviral activity of combination HIV-1 broadly neutralizing antibodies in viremic individuals. Nat. Med. 24, 1701–1707. 10.1038/s41591-018-0186-4 30258217 PMC6221973

[B7] Barrangou R. Fremaux C. Deveau H. Richards M. Boyaval P. Moineau S.. (2007). CRISPR provides acquired resistance against viruses in prokaryotes. Science 315, 1709–1712. 10.1126/science.1138140 17379808

[B8] Bialek J. K. Dunay G. A. Voges M. Schafer C. Spohn M. Stucka R.. (2016). Targeted HIV-1 latency reversal using CRISPR/Cas9-derived transcriptional activator systems. PLoS ONE 11:e0158294. 10.1371/journal.pone.0158294 27341108 PMC4920395

[B9] Biti R. Ffrench R. Young J. Bennetts B. Stewart G. Liang T. (1997). HIV-1 infection in an individual homozygous for the CCR5 deletion allele. Nat. Med. 3, 252–253. 10.1038/nm0397-252 9055842

[B10] Bogerd H. P. Kornepati A. V. Marshall J. B. Kennedy E. M. Cullen B. R. (2015). Specific induction of endogenous viral restriction factors using CRISPR/Cas-derived transcriptional activators. Proc. Natl. Acad. Sci. U.S.A. 112, E7249–56. 10.1073/pnas.1516305112 26668372 PMC4703010

[B11] Bowers N. L. Helton E. S. Huijbregts R. P. Goepfert P. A. Heath S. L. Hel Z. (2014). Immune suppression by neutrophils in HIV-1 infection: role of PD-L1/PD-1 pathway. PLoS Pathog. 10:e1003993. 10.1371/journal.ppat.1003993 24626392 PMC3953441

[B12] Bullen C. K. Laird G. M. Durand C. M. Siliciano J. D. Siliciano R. F. (2014). New ex vivo approaches distinguish effective and ineffective single agents for reversing HIV-1 latency *in vivo*. Nat. Med. 20, 425–429. 10.1038/nm.3489 24658076 PMC3981911

[B13] Cardarelli F. Digiacomo L. Marchini C. Amici A. Salomone F. Fiume G.. (2016). The intracellular trafficking mechanism of Lipofectamine-based transfection reagents and its implication for gene delivery. Sci. Rep. 6:25879. 10.1038/srep25879 27165510 PMC4863168

[B14] Cassan E. Arigon-Chifolleau A. M. Mesnard J. M. Gross A. Gascuel O. (2016). Concomitant emergence of the antisense protein gene of HIV-1 and of the pandemic. Proc. Natl. Acad. Sci. U.S.A. 113, 11537–11542. 10.1073/pnas.1605739113 27681623 PMC5068275

[B15] Charlesworth C. T. Deshpande P. S. Dever D. P. Camarena J. Lemgart V. T. Cromer M. K.. (2019). Identification of preexisting adaptive immunity to Cas9 proteins in humans. Nat. Med. 25, 249–254. 10.1038/s41591-018-0326-x 30692695 PMC7199589

[B16] Chavez L. Calvanese V. Verdin E. (2015). HIV latency is established directly and early in both resting and activated primary CD4 T cells. PLoS Pathog. 11:e1004955. 10.1371/journal.ppat.1004955 26067822 PMC4466167

[B17] Chemudupati M. Kenney A. D. Bonifati S. Zani A. McMichael T. M. Wu L.. (2019). From APOBEC to ZAP: diverse mechanisms used by cellular restriction factors to inhibit virus infections. Biochim. Biophys. Acta Mol. Cell Res. 1866, 382–394. 10.1016/j.bbamcr.2018.09.012 30290238 PMC6334645

[B18] Chen S. Yu X. Guo D. (2018). CRISPR-Cas targeting of host genes as an antiviral strategy. Viruses 10:E40. 10.3390/v10010040 29337866 PMC5795453

[B19] Cho S. W. Kim S. Kim J. M. Kim J. S. (2013). Targeted genome engineering in human cells with the Cas9 RNA-guided endonuclease. Nat. Biotechnol. 31, 230–232. 10.1038/nbt.2507 23360966

[B20] Chougui G. Munir-Matloob S. Matkovic R. Martin M. M. Morel M. Lahouassa H.. (2018). HIV-2/SIV viral protein X counteracts HUSH repressor complex. Nat. Microbiol. 3, 891–897. 10.1038/s41564-018-0179-6 29891865

[B21] Chun T. W. Carruth L. Finzi D. Shen X. DiGiuseppe J. A. Taylor H.. (1997a). Quantification of latent tissue reservoirs and total body viral load in HIV-1 infection. Nature 387, 183–188. 10.1038/387183a0 9144289

[B22] Chun T. W. Engel D. Mizell S. B. Ehler L. A. Fauci A. S. (1998). Induction of HIV-1 replication in latently infected CD4+ T cells using a combination of cytokines. J. Exp. Med. 188, 83–91. 10.1084/jem.188.1.83 9653086 PMC2525548

[B23] Chun T. W. Nickle D. C. Justement J. S. Meyers J. H. Roby G. Hallahan C. W.. (2008). Persistence of HIV in gut-associated lymphoid tissue despite long-term antiretroviral therapy. J. Infect. Dis. 197, 714–720. 10.1086/527324 18260759

[B24] Chun T. W. Stuyver L. Mizell S. B. Ehler L. A. Mican J. A. Baseler M.. (1997b). Presence of an inducible HIV-1 latent reservoir during highly active antiretroviral therapy. Proc. Natl. Acad. Sci. U.S.A. 94, 13193–13197. 10.1073/pnas.94.24.13193 9371822 PMC24285

[B25] Chung S. H. Seki K. Choi B. I. Kimura K. B. Ito A. Fujikado N.. (2010). CXC chemokine receptor 4 expressed in T cells plays an important role in the development of collagen-induced arthritis. Arthritis Res. Ther. 12:R188. 10.1186/ar3158 20939892 PMC2991023

[B26] Churchill M. J. Gorry P. R. Cowley D. Lal L. Sonza S. Purcell D. F.. (2006). Use of laser capture microdissection to detect integrated HIV-1 DNA in macrophages and astrocytes from autopsy brain tissues. J. Neurovirol. 12, 146–152. 10.1080/13550280600748946 16798676

[B27] Cocchi F. DeVico A. L. Garzino-Demo A. Arya S. K. Gallo R. C. Lusso P. (1995). Identification of RANTES, MIP-1 alpha, and MIP-1 beta as the major HIV-suppressive factors produced by CD8+ T cells. Science 270, 1811–1815. 10.1126/science.270.5243.1811 8525373

[B28] Cohen M. S. Shaw G. M. McMichael A. J. Haynes B. F. (2011). Acute HIV-1 infection. N. Engl. J. Med. 364, 1943–1954. 10.1056/NEJMra1011874 21591946 PMC3771113

[B29] Cong L. Ran F. A. Cox D. Lin S. Barretto R. Habib N.. (2013). Multiplex genome engineering using CRISPR/Cas systems. Science 339, 819–823. 10.1126/science.1231143 23287718 PMC3795411

[B30] Crudele J. M. Chamberlain J. S. (2018). Cas9 immunity creates challenges for CRISPR gene editing therapies. Nat. Commun. 9:3497. 10.1038/s41467-018-05843-9 30158648 PMC6115392

[B31] Cyranoski D. (2016). CRISPR gene-editing tested in a person for the first time. Nature 539:479. 10.1038/nature.2016.20988 27882996

[B32] Dar A. Kollet O. Lapidot T. (2006). Mutual, reciprocal SDF-1/CXCR4 interactions between hematopoietic and bone marrow stromal cells regulate human stem cell migration and development in NOD/SCID chimeric mice. Exp. Hematol. 34, 967–975. 10.1016/j.exphem.2006.04.002 16863903

[B33] Darcis G. Das A. T. Berkhout B. (2018). Tackling HIV persistence: pharmacological versus CRISPR-based shock strategies. Viruses 10:E157. 10.3390/v10040157 29596334 PMC5923451

[B34] De Ravin S. S. Li L. Wu X. Choi U. Allen C. Koontz S.. (2017). CRISPR-Cas9 gene repair of hematopoietic stem cells from patients with X-linked chronic granulomatous disease. Sci. Transl. Med. 9:eaah3480. 10.1126/scitranslmed.aah3480 28077679

[B35] Delagreverie H. M. Delaugerre C. Lewin S. R. Deeks S. G. Li J. Z. (2016). Ongoing clinical trials of human immunodeficiency virus latency-reversing and immunomodulatory agents. Open Forum Infect. Dis. 3:ofw189. 10.1093/ofid/ofw189 27757411 PMC5066458

[B36] Didigu C. A. Wilen C. B. Wang J. Duong J. Secreto A. J. Danet-Desnoyers G. A.. (2014). Simultaneous zinc-finger nuclease editing of the HIV coreceptors ccr5 and cxcr4 protects CD4+ T cells from HIV-1 infection. Blood 123, 61–69. 10.1182/blood-2013-08-521229 24162716 PMC3879906

[B37] du Chene I. Basyuk E. Lin Y. L. Triboulet R. Knezevich A. Chable-Bessia C.. (2007). Suv39H1 and HP1gamma are responsible for chromatin-mediated HIV-1 transcriptional silencing and post-integration latency. EMBO J. 26, 424–435. 10.1038/sj.emboj.7601517 17245432 PMC1783455

[B38] Duan J. Lu G. Xie Z. Lou M. Luo J. Guo L.. (2014). Genome-wide identification of CRISPR/Cas9 off-targets in human genome. Cell Res. 24, 1009–1012. 10.1038/cr.2014.87 24980957 PMC4123298

[B39] Ebina H. Misawa N. Kanemura Y. Koyanagi Y. (2013). Harnessing the CRISPR/Cas9 system to disrupt latent HIV-1 provirus. Sci. Rep. 3:2510. 10.1038/srep02510 23974631 PMC3752613

[B40] Fischer-Smith T. Croul S. Sverstiuk A. E. Capini C. L'Heureux D. Regulier E. G.. (2001). CNS invasion by CD14+/CD16+ peripheral blood-derived monocytes in HIV dementia: perivascular accumulation and reservoir of HIV infection. J. Neurovirol. 7, 528–541. 10.1080/135502801753248114 11704885

[B41] Fornaguera C. Dols-Perez A. Caldero G. Garcia-Celma M. J. Camarasa J. Solans C. (2015). PLGA nanoparticles prepared by nano-emulsion templating using low-energy methods as efficient nanocarriers for drug delivery across the blood-brain barrier. J. Control Release 211, 134–143. 10.1016/j.jconrel.2015.06.002 26057857

[B42] Fu Y. Sander J. D. Reyon D. Cascio V. M. Joung J. K. (2014). Improving CRISPR-Cas nuclease specificity using truncated guide RNAs. Nat. Biotechnol. 32, 279–284. 10.1038/nbt.2808 24463574 PMC3988262

[B43] Gallastegui E. Millan-Zambrano G. Terme J. M. Chavez S. Jordan A. (2011). Chromatin reassembly factors are involved in transcriptional interference promoting HIV latency. J. Virol. 85, 3187–3202. 10.1128/JVI.01920-10 21270164 PMC3067836

[B44] Gilbert L. A. Horlbeck M. A. Adamson B. Villalta J. E. Chen Y. Whitehead E. H.. (2014). Genome-scale CRISPR-mediated control of gene repression and activation. Cell 159, 647–661. 10.1016/j.cell.2014.09.029 25307932 PMC4253859

[B45] Gilbert L. A. Larson M. H. Morsut L. Liu Z. Brar G. A. Torres S. E.. (2013). CRISPR-mediated modular RNA-guided regulation of transcription in eukaryotes. Cell 154, 442–451. 10.1016/j.cell.2013.06.044 23849981 PMC3770145

[B46] Givens B. E. Naguib Y. W. Geary S. M. Devor E. J. Salem A. K. (2018). Nanoparticle-based delivery of CRISPR/Cas9 genome-editing therapeutics. AAPS J. 20:108. 10.1208/s12248-018-0267-9 30306365 PMC6398936

[B47] Glass W. G. McDermott D. H. Lim J. K. Lekhong S. Yu S. F. Frank W. A.. (2006). CCR5 deficiency increases risk of symptomatic West Nile virus infection. J. Exp. Med. 203, 35–40. 10.1084/jem.20051970 16418398 PMC2118086

[B48] Gonzalez-Enriquez G. V. Escoto-Delgadillo M. Vazquez-Valls E. Torres-Mendoza B. M. (2017). SERINC as a restriction factor to inhibit viral infectivity and the interaction with HIV. J. Immunol. Res. 2017:1548905. 10.1155/2017/1548905 29359168 PMC5735641

[B49] Gootenberg J. S. Abudayyeh O. O. Lee J. W. Essletzbichler P. Dy A. J. Joung J.. (2017). Nucleic acid detection with CRISPR-Cas13a/C2c2. Science 356, 438–442. 10.1126/science.aam9321 28408723 PMC5526198

[B50] Hill A. L. Rosenbloom D. I. Fu F. Nowak M. A. Siliciano R. F. (2014). Predicting the outcomes of treatment to eradicate the latent reservoir for HIV-1. Proc. Natl. Acad. Sci. U.S.A. 111, 13475–13480. 10.1073/pnas.1406663111 25097264 PMC4169952

[B51] Hladik F. McElrath M. J. (2008). Setting the stage: host invasion by HIV. Nat. Rev. Immunol. 8, 447–457. 10.1038/nri2302 18469831 PMC2587276

[B52] Ho Y. C. Shan L. Hosmane N. N. Wang J. Laskey S. B. Rosenbloom D. I.. (2013). Replication-competent noninduced proviruses in the latent reservoir increase barrier to HIV-1 cure. Cell 155, 540–551. 10.1016/j.cell.2013.09.020 24243014 PMC3896327

[B53] Holt N. Wang J. Kim K. Friedman G. Wang X. Taupin V.. (2010). Human hematopoietic stem/progenitor cells modified by zinc-finger nucleases targeted to CCR5 control HIV-1 *in vivo*. Nat. Biotechnol. 28, 839–847. 10.1038/nbt.1663 20601939 PMC3080757

[B54] Hou P. Chen S. Wang S. Yu X. Chen Y. Jiang M.. (2015). Genome editing of CXCR4 by CRISPR/cas9 confers cells resistant to HIV-1 infection. Sci. Rep. 5:15577. 10.1038/srep15577 26481100 PMC4612538

[B55] Hsu P. D. Lander E. S. Zhang F. (2014). Development and applications of CRISPR-Cas9 for genome engineering. Cell 157, 1262–1278. 10.1016/j.cell.2014.05.010 24906146 PMC4343198

[B56] Hu W. Kaminski R. Yang F. Zhang Y. Cosentino L. Li F.. (2014). RNA-directed gene editing specifically eradicates latent and prevents new HIV-1 infection. Proc. Natl. Acad. Sci. U.S.A. 111, 11461–11466. 10.1073/pnas.1405186111 25049410 PMC4128125

[B57] Huang J. Wang F. Argyris E. Chen K. Liang Z. Tian H.. (2007). Cellular microRNAs contribute to HIV-1 latency in resting primary CD4+ T lymphocytes. Nat. Med. 13, 1241–1247. 10.1038/nm1639 17906637

[B58] Huang S. H. Ren Y. Thomas A. S. Chan D. Mueller S. Ward A. R.. (2018). Latent HIV reservoirs exhibit inherent resistance to elimination by CD8+ T cells. J. Clin. Invest. 128, 876–889. 10.1172/JCI97555 29355843 PMC5785246

[B59] Hutter G. Nowak D. Mossner M. Ganepola S. Mussig A. Allers K.. (2009). Long-term control of HIV by CCR5 Delta32/Delta32 stem-cell transplantation. N. Engl. J. Med. 360, 692–698. 10.1056/NEJMoa0802905 19213682

[B60] Ishino Y. Shinagawa H. Makino K. Amemura M. Nakata A. (1987). Nucleotide sequence of the iap gene, responsible for alkaline phosphatase isozyme conversion in *Escherichia coli*, and identification of the gene product. J. Bacteriol. 169, 5429–5433. 10.1128/jb.169.12.5429-5433.1987 3316184 PMC213968

[B61] Ji H. Jiang Z. Lu P. Ma L. Li C. Pan H.. (2016). Specific reactivation of latent HIV-1 by dCas9-suntag-VP64-mediated guide RNA targeting the HIV-1 promoter. Mol. Ther. 24, 508–521. 10.1038/mt.2016.7 26775808 PMC4786936

[B62] Jiang S. Wen N. Li Z. Dube U. Del Aguila J. Budde J.. (2018). Integrative system biology analyses of CRISPR-edited iPSC-derived neurons and human brains reveal deficiencies of presynaptic signaling in FTLD and PSP. Transl. Psychiatry 8:265. 10.1038/s41398-018-0319-z 30546007 PMC6293323

[B63] Jinek M. Chylinski K. Fonfara I. Hauer M. Doudna J. A. Charpentier E. (2012). A programmable dual-RNA-guided DNA endonuclease in adaptive bacterial immunity. Science 337, 816–821. 10.1126/science.1225829 22745249 PMC6286148

[B64] Jinek M. Jiang F. Taylor D. W. Sternberg S. H. Kaya E. Ma E.. (2014). Structures of Cas9 endonucleases reveal RNA-mediated conformational activation. Science 343:1247997. 10.1126/science.1247997 24505130 PMC4184034

[B65] Kaminski R. Chen Y. Salkind J. Bella R. Young W. B. Ferrante P.. (2016). Negative feedback regulation of HIV-1 by gene editing strategy. Sci. Rep. 6:31527. 10.1038/srep31527 27528385 PMC4985742

[B66] Kang H. Minder P. Park M. A. Mesquitta W. T. Torbett B. E. Slukvin I. I. (2015). CCR5 disruption in induced pluripotent stem cells using CRISPR/Cas9 provides selective resistance of immune cells to CCR5-tropic HIV-1 virus. Mol. Ther. Nucleic Acids 4:e268. 10.1038/mtna.2015.42 26670276

[B67] Khalili K. Kaminski R. Gordon J. Cosentino L. Hu W. (2015). Genome editing strategies: potential tools for eradicating HIV-1/AIDS. J. Neurovirol. 21, 310–321. 10.1007/s13365-014-0308-9 25716921 PMC4433555

[B68] Khalili K. White M. K. Jacobson J. M. (2017). Novel AIDS therapies based on gene editing. Cell Mol. Life Sci. 74, 2439–50. 10.1007/s00018-017-2479-z 28210784 PMC5474186

[B69] Kim S. Koo T. Jee H. G. Cho H. Y. Lee G. Lim D. G. (2018). CRISPR RNAs trigger innate immune responses in human cells. Genome Res. 28, 367–373. 10.1101/gr.231936.117 PMC5848615 29472270

[B70] Kim V. Mears B. M. Powell B. H. Witwer K. W. (2017). Mutant Cas9-transcriptional activator activates HIV-1 in U1 cells in the presence and absence of LTR-specific guide RNAs. Matters 2017, 1–4. 10.19185/matters.201611000027 28670581 PMC5493433

[B71] Kim Y. Anderson J. L. Lewin S. R. (2018). Getting the “Kill” into “Shock and Kill”: strategies to eliminate latent HIV. Cell Host Microbe 23, 14–26. 10.1016/j.chom.2017.12.004 29324227 PMC5990418

[B72] Kimberland M. L. Hou W. Alfonso-Pecchio A. Wilson S. Rao Y. Zhang S.. (2018). Strategies for controlling CRISPR/Cas9 off-target effects and biological variations in mammalian genome editing experiments. J. Biotechnol. 284, 91–101. 10.1016/j.jbiotec.2018.08.007 30142414

[B73] Konermann S. Brigham M. D. Trevino A. E. Joung J. Abudayyeh O. O. Barcena C.. (2015). Genome-scale transcriptional activation by an engineered CRISPR-Cas9 complex. Nature 517, 583–588. 10.1038/nature14136 25494202 PMC4420636

[B74] Lahaye X. Gentili M. Silvin A. Conrad C. Picard L. Jouve M.. (2018). NONO detects the nuclear HIV capsid to promote cGAS-mediated innate immune activation. Cell 175, 488–501 e422. 10.1016/j.cell.2018.08.062 30270045

[B75] Larder B. A. Darby G. Richman D. D. (1989). HIV with reduced sensitivity to zidovudine (AZT) isolated during prolonged therapy. Science 243, 1731–1734. 10.1126/science.2467383 2467383

[B76] Lebbink R. J. de Jong D. C. Wolters F. Kruse E. M. van Ham P. M. Wiertz E. J.. (2017). A combinational CRISPR/Cas9 gene-editing approach can halt HIV replication and prevent viral escape. Sci. Rep. 7:41968. 10.1038/srep41968 28176813 PMC5296774

[B77] Lenasi T. Contreras X. Peterlin B. M. (2008). Transcriptional interference antagonizes proviral gene expression to promote HIV latency. Cell Host Microbe 4, 123–133. 10.1016/j.chom.2008.05.016 18692772 PMC4217705

[B78] Li C. Guan X. Du T. Jin W. Wu B. Liu Y.. (2015). Inhibition of HIV-1 infection of primary CD4+ T-cells by gene editing of CCR5 using adenovirus-delivered CRISPR/Cas9. J. Gen. Virol. 96, 2381–2393. 10.1099/vir.0.000139 25854553

[B79] Li L. He Z. Y. Wei X. W. Gao G. P. Wei Y. Q. (2015). Challenges in CRISPR/CAS9 delivery: potential roles of nonviral vectors. Hum. Gene Ther. 26, 452–462. 10.1089/hum.2015.069 26176432

[B80] Liang C. Wainberg M. A. Das A. T. Berkhout B. (2016). CRISPR/Cas9: a double-edged sword when used to combat HIV infection. Retrovirology 13:37. 10.1186/s12977-016-0270-0 27230886 PMC4882869

[B81] Liao H. K. Gu Y. Diaz A. Marlett J. Takahashi Y. Li M.. (2015). Use of the CRISPR/Cas9 system as an intracellular defense against HIV-1 infection in human cells. Nat. Commun. 6:6413. 10.1038/ncomms7413 25752527

[B82] Lim J. K. Louie C. Y. Glaser C. Jean C. Johnson B. Johnson H.. (2008). Genetic deficiency of chemokine receptor CCR5 is a strong risk factor for symptomatic West Nile virus infection: a meta-analysis of 4 cohorts in the US epidemic. J. Infect. Dis. 197, 262–265. 10.1086/524691 18179388

[B83] Lim K. R. Q. Yoon C. Yokota T. (2018). Applications of CRISPR/Cas9 for the treatment of duchenne muscular dystrophy. J. Pers. Med. 8:E38. 10.3390/jpm8040038 30477208 PMC6313657

[B84] Limsirichai P. Gaj T. Schaffer D. V. (2016). CRISPR-mediated activation of latent HIV-1 expression. Mol. Ther. 24, 499–507. 10.1038/mt.2015.213 26607397 PMC4786916

[B85] Liu Q. Lai Y. T. Zhang P. Louder M. K. Pegu A. Rawi R.. (2019). Improvement of antibody functionality by structure-guided paratope engraftment. Nat. Commun. 10:721. 10.1038/s41467-019-08658-4 30760721 PMC6374468

[B86] Liu S. Wang Q. Yu X. Li Y. Guo Y. Liu Z.. (2018). HIV-1 inhibition in cells with CXCR4 mutant genome created by CRISPR-Cas9 and piggyBac recombinant technologies. Sci. Rep. 8:8573. 10.1038/s41598-018-26894-4 29872154 PMC5988798

[B87] Liu Z. Chen S. Jin X. Wang Q. Yang K. Li C.. (2017). Genome editing of the HIV co-receptors CCR5 and CXCR4 by CRISPR-Cas9 protects CD4(+) T cells from HIV-1 infection. Cell Biosci. 7:47. 10.1186/s13578-017-0174-2 28904745 PMC5591563

[B88] Liu Z. Torresilla C. Xiao Y. Nguyen P. T. Cate C. Barbosa K.. (2018). HIV-1 antisense protein of different clades induces autophagy and associates to the autophagy factor p62. J. Virol. 93:e01757-18. 10.1128/JVI.01757-18 30404795 PMC6321906

[B89] Lorenzo-Redondo R. Fryer H. R. Bedford T. Kim E. Y. Archer J. Pond S. L. K.. (2016). Persistent HIV-1 replication maintains the tissue reservoir during therapy. Nature 530, 51–56. 10.1038/nature16933 26814962 PMC4865637

[B90] Louis Jeune V. Joergensen J. A. Hajjar R. J. Weber T. (2013). Pre-existing anti-adeno-associated virus antibodies as a challenge in AAV gene therapy. Hum. Gene Ther. Methods 24, 59–67. 10.1089/hgtb.2012.243 23442094 PMC3732124

[B91] Mangeot P. E. Risson V. Fusil F. Marnef A. Laurent E. Blin J.. (2019). Genome editing in primary cells and *in vivo* using viral-derived Nanoblades loaded with Cas9-sgRNA ribonucleoproteins. Nat. Commun. 10:45. 10.1038/s41467-018-07845-z 30604748 PMC6318322

[B92] Manson McManamy M. E. Hakre S. Verdin E. M. Margolis D. M. (2014). Therapy for latent HIV-1 infection: the role of histone deacetylase inhibitors. Antivir. Chem. Chemother. 23, 145–149. 10.3851/IMP2551 24318952 PMC3947511

[B93] Mefferd A. L. Bogerd H. P. Irwan I. D. Cullen B. R. (2018). Insights into the mechanisms underlying the inactivation of HIV-1 proviruses by CRISPR/Cas. Virology 520, 116–126. 10.1016/j.virol.2018.05.016 29857168 PMC6100742

[B94] Mendoza P. Gruell H. Nogueira L. Pai J. A. Butler A. L. Millard K.. (2018). Combination therapy with anti-HIV-1 antibodies maintains viral suppression. Nature 561, 479–484. 10.1038/s41586-018-0531-2 30258136 PMC6166473

[B95] Michael N. L. Chang G. Louie L. G. Mascola J. R. Dondero D. Birx D. L.. (1997). The role of viral phenotype and CCR-5 gene defects in HIV-1 transmission and disease progression. Nat. Med. 3, 338–340. 10.1038/nm0397-338 9055864

[B96] Mingozzi F. High K. A. (2013). Immune responses to AAV vectors: overcoming barriers to successful gene therapy. Blood 122, 23–36. 10.1182/blood-2013-01-306647 23596044 PMC3701904

[B97] Mingozzi F. Meulenberg J. J. Hui D. J. Basner-Tschakarjan E. Hasbrouck N. C. Edmonson S. A.. (2009). AAV-1-mediated gene transfer to skeletal muscle in humans results in dose-dependent activation of capsid-specific T cells. Blood 114, 2077–2086. 10.1182/blood-2008-07-167510 19506302 PMC2744569

[B98] Mojica F. J. Diez-Villasenor C. Garcia-Martinez J. Almendros C. (2009). Short motif sequences determine the targets of the prokaryotic CRISPR defence system. Microbiology 155(Pt 3), 733–740. 10.1099/mic.0.023960-0 19246744

[B99] Mojica F. J. Diez-Villasenor C. Garcia-Martinez J. Soria E. (2005). Intervening sequences of regularly spaced prokaryotic repeats derive from foreign genetic elements. J. Mol. Evol. 60, 174–182. 10.1007/s00239-004-0046-3 15791728

[B100] Mout R. Ray M. Yesilbag Tonga G. Lee Y. W. Tay T. Sasaki K.. (2017). Direct cytosolic delivery of CRISPR/Cas9-ribonucleoprotein for efficient gene editing. ACS Nano. 11, 2452–2458. 10.1021/acsnano.6b07600 28129503 PMC5848212

[B101] Nagasawa T. Nakajima T. Tachibana K. Iizasa H. Bleul C. C. Yoshie O.. (1996). Molecular cloning and characterization of a murine pre-B-cell growth-stimulating factor/stromal cell-derived factor 1 receptor, a murine homolog of the human immunodeficiency virus 1 entry coreceptor fusin. Proc. Natl. Acad. Sci. U.S.A. 93, 14726–14729. 10.1073/pnas.93.25.14726 8962122 PMC26203

[B102] Nair M. Guduru R. Liang P. Hong J. Sagar V. Khizroev S. (2013). Externally controlled on-demand release of anti-HIV drug using magneto-electric nanoparticles as carriers. Nat. Commun. 4:1707. 10.1038/ncomms2717 23591874

[B103] Narasipura S. D. Kim S. Al-Harthi L. (2014). Epigenetic regulation of HIV-1 latency in astrocytes. J. Virol. 88, 3031–3038. 10.1128/JVI.03333-13 24352441 PMC3958059

[B104] Nath A. (2015). Eradication of human immunodeficiency virus from brain reservoirs. J. Neurovirol. 21, 227–234. 10.1007/s13365-014-0291-1 25366659 PMC4418952

[B105] Niethammer M. Tang C. C. Vo A. Nguyen N. Spetsieris P. Dhawan V.. (2018). Gene therapy reduces Parkinson's disease symptoms by reorganizing functional brain connectivity. Sci. Transl. Med. 10:eaau0713. 10.1126/scitranslmed.aau0713 30487248

[B106] Nishimasu H. Ran F. A. Hsu P. D. Konermann S. Shehata S. I. Dohmae N.. (2014). Crystal structure of Cas9 in complex with guide RNA and target DNA. Cell 156, 935–949. 10.1016/j.cell.2014.02.001 24529477 PMC4139937

[B107] Nyamweya S. Hegedus A. Jaye A. Rowland-Jones S. Flanagan K. L. Macallan D. C. (2013). Comparing HIV-1 and HIV-2 infection: lessons for viral immunopathogenesis. Rev. Med. Virol. 23, 221–240. 10.1002/rmv.1739 23444290

[B108] Palella F. J. Jr. Delaney K. M. Moorman A. C. Loveless M. O. Fuhrer J. Satten G. A.. (1998). Declining morbidity and mortality among patients with advanced human immunodeficiency virus infection. HIV outpatient study investigators. N. Engl. J. Med. 338, 853–860. 10.1056/NEJM199803263381301 9516219

[B109] Panfil A. R. London J. A. Green P. L. Yoder K. E. (2018). CRISPR/Cas9 genome editing to disable the latent HIV-1 provirus. Front. Microbiol. 9:3107. 10.3389/fmicb.2018.03107 30619186 PMC6302043

[B110] Patel P. Ansari M. Y. Bapat S. Thakar M. Gangakhedkar R. Jameel S. (2014). The microRNA miR-29a is associated with human immunodeficiency virus latency. Retrovirology 11:108. 10.1186/s12977-014-0108-6 25486977 PMC4269869

[B111] Perez E. E. Wang J. Miller J. C. Jouvenot Y. Kim K. A. Liu O.. (2008). Establishment of HIV-1 resistance in CD4+ T cells by genome editing using zinc-finger nucleases. Nat. Biotechnol. 26, 808–816. 10.1038/nbt1410 18587387 PMC3422503

[B112] Qi C. Li D. Jiang X. Jia X. Lu L. Wang Y.. (2018). Inducing CCR5Delta32/Delta32 homozygotes in the human jurkat CD4+ cell line and primary CD4+ cells by CRISPR-Cas9 genome-editing technology. Mol. Ther. Nucleic Acids 12, 267–274. 10.1016/j.omtn.2018.05.012 30195765 PMC6005807

[B113] Qi L. S. Larson M. H. Gilbert L. A. Doudna J. A. Weissman J. S. Arkin A. P.. (2013). Repurposing CRISPR as an RNA-guided platform for sequence-specific control of gene expression. Cell 152, 1173–1183. 10.1016/j.cell.2013.02.022 23452860 PMC3664290

[B114] Qu X. Wang P. Ding D. Li L. Wang H. Ma L.. (2013). Zinc-finger-nucleases mediate specific and efficient excision of HIV-1 proviral DNA from infected and latently infected human T cells. Nucleic Acids Res. 41, 7771–7782. 10.1093/nar/gkt571 23804764 PMC3763554

[B115] Ran F. A. Hsu P. D. Lin C. Y. Gootenberg J. S. Konermann S. Trevino A. E.. (2013a). Double nicking by RNA-guided CRISPR Cas9 for enhanced genome editing specificity. Cell 154, 1380–1389. 10.1016/j.cell.2013.08.021 23992846 PMC3856256

[B116] Ran F. A. Hsu P. D. Wright J. Agarwala V. Scott D. A. Zhang F. (2013b). Genome engineering using the CRISPR-Cas9 system. Nat. Protoc. 8, 2281–2308. 10.1038/nprot.2013.143 24157548 PMC3969860

[B117] Rasmussen T. A. Tolstrup M. Brinkmann C. R. Olesen R. Erikstrup C. Solomon A.. (2014). Panobinostat, a histone deacetylase inhibitor, for latent-virus reactivation in HIV-infected patients on suppressive antiretroviral therapy: a phase 1/2, single group, clinical trial. Lancet HIV 1, e13–21. 10.1016/S2352-3018(14)70014-1 26423811

[B118] Rosa A. Chande A. Ziglio S. De Sanctis V. Bertorelli R. Goh S. L.. (2015). HIV-1 Nef promotes infection by excluding SERINC5 from virion incorporation. Nature 526, 212–217. 10.1038/nature15399 26416734 PMC4861059

[B119] Ruelas D. S. Chan J. K. Oh E. Heidersbach A. J. Hebbeler A. M. Chavez L.. (2015). MicroRNA-155 reinforces HIV latency. J. Biol. Chem. 290, 13736–13748. 10.1074/jbc.M115.641837 25873391 PMC4447952

[B120] Saayman S. M. Lazar D. C. Scott T. A. Hart J. R. Takahashi M. Burnett J. C.. (2016). Potent and targeted activation of latent HIV-1 using the CRISPR/dCas9 activator complex. Mol. Ther. 24, 488–498. 10.1038/mt.2015.202 26581162 PMC4786915

[B121] Samson M. Libert F. Doranz B. J. Rucker J. Liesnard C. Farber C. M.. (1996). Resistance to HIV-1 infection in caucasian individuals bearing mutant alleles of the CCR-5 chemokine receptor gene. Nature 382, 722–725. 10.1038/382722a0 8751444

[B122] Sander J. D. Joung J. K. (2014). CRISPR-Cas systems for editing, regulating and targeting genomes. Nat. Biotechnol. 32, 347–355. 10.1038/nbt.2842 24584096 PMC4022601

[B123] Sarkar I. Hauber I. Hauber J. Buchholz F. (2007). HIV-1 proviral DNA excision using an evolved recombinase. Science 316, 1912–1915. 10.1126/science.1141453 17600219

[B124] Schumann K. Lin S. Boyer E. Simeonov D. R. Subramaniam M. Gate R. E.. (2015). Generation of knock-in primary human T cells using Cas9 ribonucleoproteins. Proc. Natl. Acad. Sci. U.S.A. 112, 10437–10442. 10.1073/pnas.1512503112 26216948 PMC4547290

[B125] Sharp P. M. Hahn B. H. (2011). Origins of HIV and the AIDS pandemic. Cold Spring Harb. Perspect. Med. 1:a006841. 10.1101/cshperspect.a006841 22229120 PMC3234451

[B126] Shen B. Zhang W. Zhang J. Zhou J. Wang J. Chen L.. (2014). Efficient genome modification by CRISPR-Cas9 nickase with minimal off-target effects. Nat. Methods 11, 399–402. 10.1038/nmeth.2857 24584192

[B127] Siliciano J. D. Kajdas J. Finzi D. Quinn T. C. Chadwick K. Margolick J. B.. (2003). Long-term follow-up studies confirm the stability of the latent reservoir for HIV-1 in resting CD4+ T cells. Nat. Med. 9, 727–728. 10.1038/nm880 12754504

[B128] Smith P. D. Meng G. Salazar-Gonzalez J. F. Shaw G. M. (2003). Macrophage HIV-1 infection and the gastrointestinal tract reservoir. J. Leukoc. Biol. 74, 642–649. 10.1189/jlb.0503219 12960227

[B129] Sunshine S. Kirchner R. Amr S. S. Mansur L. Shakhbatyan R. Kim M.. (2016). HIV integration site analysis of cellular models of HIV latency with a probe-enriched next-generation sequencing assay. J. Virol. 90, 4511–4519. 10.1128/JVI.01617-15 26912621 PMC4836361

[B130] Tebas P. Stein D. Tang W. W. Frank I. Wang S. Q. Lee G.. (2014). Gene editing of CCR5 in autologous CD4 T cells of persons infected with HIV. N. Engl. J. Med. 370, 901–910. 10.1056/NEJMoa1300662 24597865 PMC4084652

[B131] Tsai S. Q. Wyvekens N. Khayter C. Foden J. A. Thapar V. Reyon D.. (2014). Dimeric CRISPR RNA-guided FokI nucleases for highly specific genome editing. Nat. Biotechnol. 32, 569–576. 10.1038/nbt.2908 24770325 PMC4090141

[B132] Vakulskas C. A. Dever D. P. Rettig G. R. Turk R. Jacobi A. M. Collingwood M. A.. (2018). A high-fidelity Cas9 mutant delivered as a ribonucleoprotein complex enables efficient gene editing in human hematopoietic stem and progenitor cells. Nat. Med. 24, 1216–1224. 10.1038/s41591-018-0137-0 30082871 PMC6107069

[B133] Walker-Sperling V. E. Pohlmeyer C. W. Tarwater P. M. Blankson J. N. (2016). The effect of latency reversal agents on primary CD8+ T cells: implications for shock and kill strategies for human immunodeficiency virus eradication. EBio Med. 8, 217–229. 10.1016/j.ebiom.2016.04.019 27428432 PMC4919475

[B134] Wang F. X. Huang J. Zhang H. Ma X. Zhang H. (2008). APOBEC3G upregulation by alpha interferon restricts human immunodeficiency virus type 1 infection in human peripheral plasmacytoid dendritic cells. J. Gen. Virol. 89 (Pt 3), 722–730. 10.1099/vir.0.83530-0 18272764

[B135] Wang G. Zhao N. Berkhout B. Das A. T. (2016a). A combinatorial CRISPR-Cas9 attack on HIV-1 DNA extinguishes all infectious provirus in infected T cell cultures. Cell Rep. 17, 2819–2826. 10.1016/j.celrep.2016.11.057 27974196

[B136] Wang G. Zhao N. Berkhout B. Das A. T. (2016b). CRISPR-Cas9 can inhibit HIV-1 replication but NHEJ repair facilitates virus escape. Mol. Ther. 24, 522–526. 10.1038/mt.2016.24 26796669 PMC4786927

[B137] Wang G. Zhao N. Berkhout B. Das A. T. (2018). CRISPR-Cas based antiviral strategies against HIV-1. Virus Res. 244, 321–332. 10.1016/j.virusres.2017.07.020 28760348

[B138] Wang Q. Chen S. Xiao Q. Liu Z. Liu S. Hou P.. (2017). Genome modification of CXCR4 by *Staphylococcus aureus* Cas9 renders cells resistance to HIV-1 infection. Retrovirology 14:51. 10.1186/s12977-017-0375-0 29141633 PMC5688617

[B139] Wang Q. Liu S. Liu Z. Ke Z. Li C. Yu X.. (2018). Genome scale screening identification of SaCas9/gRNAs for targeting HIV-1 provirus and suppression of HIV-1 infection. Virus Res. 250, 21–30. 10.1016/j.virusres.2018.04.002 29625148

[B140] Wang W. Ye C. Liu J. Zhang D. Kimata J. T. Zhou P. (2014). CCR5 gene disruption via lentiviral vectors expressing Cas9 and single guided RNA renders cells resistant to HIV-1 infection. PLoS ONE 9:e115987. 10.1371/journal.pone.0115987 25541967 PMC4277423

[B141] Wang X. Wang Y. Wu X. Wang J. Wang Y. Qiu Z.. (2015). Unbiased detection of off-target cleavage by CRISPR-Cas9 and TALENs using integrase-defective lentiviral vectors. Nat. Biotechnol. 33, 175–178. 10.1038/nbt.3127 25599175

[B142] Wang Z. Pan Q. Gendron P. Zhu W. Guo F. Cen S.. (2016). CRISPR/Cas9-derived mutations both inhibit HIV-1 replication and accelerate viral escape. Cell Rep. 15, 481–489. 10.1016/j.celrep.2016.03.042 27068471

[B143] Wilen C. B. Wang J. Tilton J. C. Miller J. C. Kim K. A. Rebar E. J.. (2011). Engineering HIV-resistant human CD4+ T cells with CXCR4-specific zinc-finger nucleases. PLoS Pathog. 7:e1002020. 10.1371/journal.ppat.1002020 21533216 PMC3077364

[B144] Wold W. S. Toth K. (2013). Adenovirus vectors for gene therapy, vaccination and cancer gene therapy. Curr. Gene Ther. 13, 421–433. 10.2174/1566523213666131125095046 24279313 PMC4507798

[B145] Xu L. Yang H. Gao Y. Chen Z. Xie L. Liu Y.. (2017). CRISPR/Cas9-Mediated CCR5 ablation in human hematopoietic stem/progenitor cells confers HIV-1 resistance *in vivo*. Mol. Ther. 25, 1782–1789. 10.1016/j.ymthe.2017.04.027 28527722 PMC5542791

[B146] Xue H. Y. Ji L. J. Gao A. M. Liu P. He J. D. Lu X. J. (2016). CRISPR-Cas9 for medical genetic screens: applications and future perspectives. J. Med. Genet. 53, 91–97. 10.1136/jmedgenet-2015-103409 26673779

[B147] Ye L. Wang J. Beyer A. I. Teque F. Cradick T. J. Qi Z.. (2014). Seamless modification of wild-type induced pluripotent stem cells to the natural CCR5Delta32 mutation confers resistance to HIV infection. Proc. Natl. Acad. Sci. U.S.A. 111, 9591–9596. 10.1073/pnas.1407473111 24927590 PMC4084478

[B148] Yin C. Zhang T. Qu X. Zhang Y. Putatunda R. Xiao X.. (2017). *In vivo* excision of HIV-1 provirus by saCas9 and multiplex single-guide RNAs in animal models. Mol. Ther. 25, 1168–1186. 10.1016/j.ymthe.2017.03.012 28366764 PMC5417847

[B149] Yin H. Song C. Q. Dorkin J. R. Zhu L. J. Li Y. Wu Q.. (2016). Therapeutic genome editing by combined viral and non-viral delivery of CRISPR system components *in vivo*. Nat. Biotechnol. 34, 328–333. 10.1038/nbt.3471 26829318 PMC5423356

[B150] Yin L. Hu S. Mei S. Sun H. Xu F. Li J.. (2018). CRISPR/Cas9 inhibits multiple steps of HIV-1 infection. Hum. Gene Ther. 29, 1264–1276. 10.1089/hum.2018.018 29644868

[B151] Yoder K. E. Bundschuh R. (2016). Host double strand break repair generates HIV-1 strains resistant to CRISPR/Cas9. Sci. Rep. 6:29530. 10.1038/srep29530 27404981 PMC4941621

[B152] Yu A. Q. Ding Y. Lu Z. Y. Hao Y. Z. Teng Z. P. Yan S. R.. (2018). TALENs-mediated homozygous CCR5Delta32 mutations endow CD4+ U87 cells with resistance against HIV1 infection. Mol. Med. Rep. 17, 243–249. 10.3892/mmr.2017.7889 29115572 PMC5780131

[B153] Yuan J. Wang J. Crain K. Fearns C. Kim K. A. Hua K. L.. (2012). Zinc-finger nuclease editing of human cxcr4 promotes HIV-1 CD4(+) T cell resistance and enrichment. Mol. Ther. 20, 849–859. 10.1038/mt.2011.310 22273578 PMC3321595

[B154] Yurkovetskiy L. Guney M. H. Kim K. Goh S. L. McCauley S. Dauphin A.. (2018). Primate immunodeficiency virus proteins Vpx and Vpr counteract transcriptional repression of proviruses by the HUSH complex. Nat. Microbiol. 3, 1354–1361. 10.1038/s41564-018-0256-x 30297740 PMC6258279

[B155] Zaiss A. K. Muruve D. A. (2008). Immunity to adeno-associated virus vectors in animals and humans: a continued challenge. Gene Ther. 15, 808–816. 10.1038/gt.2008.54 18385765

[B156] Zetsche B. Gootenberg J. S. Abudayyeh O. O. Slaymaker I. M. Makarova K. S. Essletzbichler P.. (2015). Cpf1 is a single RNA-guided endonuclease of a class 2 CRISPR-Cas system. Cell 163, 759–771. 10.1016/j.cell.2015.09.038 26422227 PMC4638220

[B157] Zhang Y. Yin C. Zhang T. Li F. Yang W. Kaminski R.. (2015). CRISPR/gRNA-directed synergistic activation mediator (SAM) induces specific, persistent and robust reactivation of the HIV-1 latent reservoirs. Sci. Rep. 5:16277. 10.1038/srep16277 26538064 PMC4633726

[B158] Zhou W. Ma D. Sun A. X. Tran H. D. Ma D. L. Singh B. K.. (2018). PD-linked CHCHD2 mutations impair CHCHD10 and MICOS complex leading to mitochondria dysfunction. Hum. Mol. Genet. 28, 1100–1116. 10.1093/hmg/ddy413 30496485 PMC6423417

[B159] Zhu W. Lei R. Le Duff Y. Li J. Guo F. Wainberg M. A.. (2015). The CRISPR/Cas9 system inactivates latent HIV-1 proviral DNA. Retrovirology 12:22. 10.1186/s12977-015-0150-z 25808449 PMC4359768

[B160] Zimmerman P. A. Buckler-White A. Alkhatib G. Spalding T. Kubofcik J. Combadiere C.. (1997). Inherited resistance to HIV-1 conferred by an inactivating mutation in CC chemokine receptor 5: studies in populations with contrasting clinical phenotypes, defined racial background, and quantified risk. Mol. Med. 3, 23–36. 10.1007/BF03401665 9132277 PMC2230106

